# *Enterococcus* Species: Multifaceted Probiotic Potential and Safety Considerations

**DOI:** 10.3390/microorganisms14040815

**Published:** 2026-04-02

**Authors:** Ojonugwa Precious John, Kayode Olayinka Afolabi, Anayochukwu Chibuike Ngene, Williams Omotola Tanimowo, Mary Ayobami Adewoyin, Michael Bamitale Osho, Rine Christopher Reuben

**Affiliations:** 1 Department of Biological Sciences, Anchor University, Lagos 102213, Nigeria; 2 Anchor University Centre for Global Health, Anchor University, Lagos 102213, Nigeria; 3 Department of Microbiology, College of Natural Sciences, Michael Okpara University of Agriculture, Umudike 440101, Abia State, Nigeria; 4 Department of Microbiology and Biotechnology, McPherson University, Seriki Sotayo 110117, Ogun State, Nigeria; 5 Department of Biology, Stephen F. Austin State University, SFA Station, Nacogdoches, TX 75962, USA

**Keywords:** agrifood system, bacteriocin, public health, pathogenicity, antimicrobial resistance

## Abstract

*Enterococcus* spp. are common but not predominant commensal bacteria that inhabit the gastrointestinal tracts of humans and animals and are widely distributed in various environmental matrices and diverse food sources. Multiple strains of beneficial enterococci are increasingly utilized as protective cultures, alternatives to antibiotics, and probiotics for controlling pathogens, mitigating disease, modulating the microbiome, and supporting overall host health. They also support food fermentation and safety, enhance sensory properties, and produce bioactive compounds such as bacteriocins with strong pathogen-inhibitory activity and multifarious health benefits. Despite their advantages in health and agrifood systems, their association with healthcare-associated infections and the spread of antimicrobial resistance raises concerns about their safety. These risks underscore the need for stringent safety evaluations before their use as probiotics, alternatives to antibiotics, or protective cultures. Here, we provide a comprehensive atlas of the multifaceted probiotic and antimicrobial potential, as well as safety considerations of beneficial enterococci. This would provide a valuable resource for future research, regulatory assessments, and applications in the agrifood system and healthcare.

## 1. Introduction

Antimicrobial resistance (AMR) has emerged as a silent pandemic, undermining the efficacy of life-saving treatments and increasing healthcare burdens worldwide. The World Health Organization (WHO) attributes this crisis primarily to the widespread overuse of antibiotics in both clinical and agricultural settings [1]. If left unaddressed, AMR-related deaths could exceed 10 million annually within the coming decades, surpassing mortality rates from major infectious diseases [2].

As conventional antibiotics lose their effectiveness, alternative strategies, such as plant-derived compounds, bacteriophages, and probiotics, have gained increasing attention as potential countermeasures against drug-resistant infections [3,4]. Probiotics, defined as viable microbial organisms that confer significant health and nutritional advantages when consumed in sufficient quantities, are particularly promising. Among probiotic bacteria, some strains of *Enterococcus* spp., a subgroup of lactic acid bacteria (LAB), exhibit unique characteristics: they enhance traditional fermented foods by improving flavor, aroma, and texture, while also producing bacteriocins, bioactive peptides that exhibit potent activity against a broad spectrum of pathogens [5,6]. This functionality suggests their potential application as both natural food preservatives and alternatives to antibiotics. However, safety concerns associated with their opportunistic pathogenicity and classification among the ESKAPE (*Enterococcus faecium*, *Staphylococcus aureus*, *Klebsiella pneumoniae*, *Acinetobacter baumannii*, *Pseudomonas aeruginosa*, and *Enterobacter* species), pathogens globally known for their ability to develop resistance to multiple antibiotics, pose a significant threat in healthcare settings and are a major cause of hospital-acquired infections [7].

The safety concerns arise from the remarkable adaptability of some enterococci to hostile environments, including antibiotic-rich conditions, which increases their pathogenic potential, particularly in some *E. faecalis* and *E. faecium* strains [8,9]. The growing antibiotic resistance has positioned some enterococci as a significant contributor to the antimicrobial resistance (AMR) crisis [10]. Virulence factors such as biofilm formation, host cell adhesion, and immune evasion mechanisms further complicate their role, making them problematic pathogens in healthcare settings. These traits explain their involvement in severe bloodstream, heart valve, and urinary tract infections [11].

The *Enterococcus* genus exhibits strain-specific heterogeneity and environmental influences that significantly affect its pathogenicity and virulence. While clinical isolates are a major concern, several studies have identified certain strains of *Enterococcus* from fermented foods that carry drug resistance genes [12,13]. These environmental differences contribute to varying resistance profiles, with clinical isolates often displaying strong resistance patterns, whereas isolates from fermented foods generally exhibit weaker resistance [14,15]. Nonetheless, even antimicrobial-resistant *Enterococcus* strains with weak resistance can pose significant risks to immunosuppressed individuals. Such infections may lead to systemic complications and severely limit the choice of effective antibiotics, raising safety concerns about using some *Enterococcus* strains from food sources as probiotics in both food and healthcare settings [16]. Consequently, rigorous evaluation of the probiotic properties of beneficial enterococci, particularly regarding safety, is essential before their application in food systems. To address these concerns, regulatory authorities, including the European Food Safety Authority (EFSA), have implemented rigorous protocols for evaluating the safety of beneficial strains of *Enterococcus* spp. These evaluations aim to ensure that probiotic enterococci intended for use in humans or animals do not pose public health risks because of their carriage of antimicrobial and virulence determinants [16]. *Enterococcus* spp. have not yet achieved Generally Recognized As Safe (GRAS) or Qualified Presumption of Safety (QPS) status [10]. Therefore, the safety of beneficial (probiotic) enterococci is assessed on a strain-by-strain basis, rather than by species. This is because while some strains are considered beneficial and harmless, others are virulent and resistant to multiple antibiotics. Notwithstanding these concerns, certain commercially available probiotic strains, such as *E. faecium* SF68, *E. lactis* DSM 7134, and *E. faecium* 669, have exhibited substantiated therapeutic efficacy and established themselves as important contributors to the probiotic market [17,18].

The paradoxical role of *Enterococcus* in food, health, and disease has sparked significant scientific debate. This review critically explores the potential of beneficial enterococci as probiotics while carefully considering their associated safety concerns. This narrative review aims to provide a comprehensive and critical analysis of the paradoxical behavior of enterococci, which could guide future applications and research, particularly in the areas of antimicrobial resistance, biotechnology, probiotic use, health, and agrifood systems.

Additionally, a comprehensive literature search was conducted across Google Scholar, Web of Science, and PubMed in April 2025 to identify and select relevant articles that highlighted multifaceted probiotic potential in agrifood and health systems. The keyword terms used included ‘*Enterococcus*’ OR ‘enterococci’ OR ‘probiotic *Enterococcus*’ OR ‘*Enterococcus* antimicrobial resistance’ OR ‘*Enterococcus* pathogenicity’ OR ‘*Enterococcus* virulence’ OR ‘*Enterococcus* nosocomial infections’ OR ‘*Enterococcus* fermentation’ OR ‘*Enterococcus* food safety’ OR ‘*Enterococcus* bacteriocin’. Articles published in the English language were included after an independent review of titles and abstracts by authors. Other enterococci-related publications outside the scope of this study, as well as editorials, meta-analyses, commentaries, and perspectives, were excluded.

## 2. Ecological Distribution of *Enterococcus*

**Figure 1:**
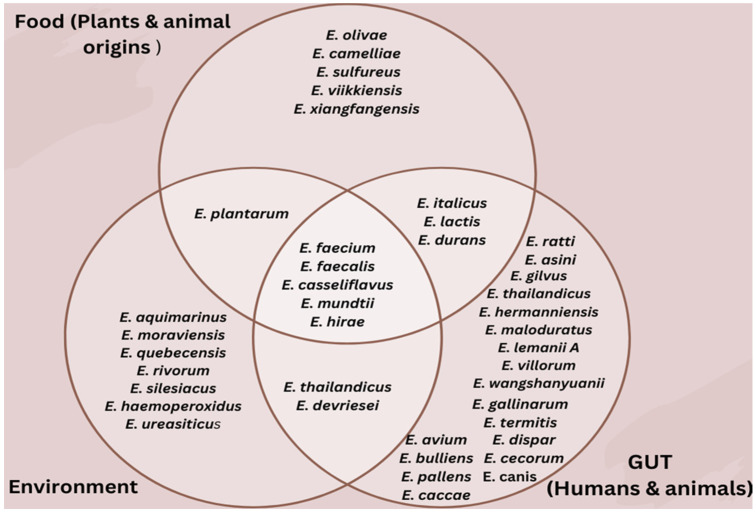
Distribution of enterococci across multiple niches.

The ecological shift of *Enterococcus* from a historic water-based environment to a land-based environment has facilitated its adaptation and ability to thrive across multiple ecological niches [19,20,21,22,23,24,25]. This adaptability has enabled enterococci to flourish in diverse environments, ranging from natural ecosystems to human-associated settings, highlighting their remarkable ecological versatility [21,22]. However, this widespread distribution also raises concerns, as some enterococci can transition from beneficial commensals to opportunistic pathogens, particularly in clinical and food-related systems (Figure 1).

### 2.1. The Gut and Nasal Cavity

The intestinal tract is a multiplex network of diverse microbes that profoundly impact human and animal health. Some strains of enterococci are common but not dominant commensals of the gastrointestinal tract of animals, and they are highly adapted to the gut ecosystem, where they thrive on abundant nutrients and interact with other microorganisms and host cells [23]. Their resilience in extreme habitats, such as low pH, high bile salt concentrations, and limited oxygen, allows them to flourish in diverse hosts [24]. In the human gastrointestinal tract, *E. faecalis* and *E. faecium* constitute the predominant species, with others such as *E. durans*, *E. hirae*, and *E. gallinarum* present at much lower abundances. They contribute to nutrient metabolism and help maintain microbial stability [25].

In animals, the composition of enterococcal species varies widely and is influenced by factors such as diet, gut physiology, and environmental exposure. While *E. faecium*, *E. faecalis*, and *E. hirae* are commonly found in the gastrointestinal tracts of cattle, pigs, and sheep, other species also exhibit host specificity. In poultry, such as chickens and turkeys, *E. faecium* and *E. durans* tend to dominate [23]. Companion animals, especially dogs, often harbor *E. faecalis*, *E. faecium*, and *E. canis* [26]; however, wild birds and rodents are frequently associated with less-characterized species such as *E. casseliflavus*, *E. avium*, *E. cecorum*, and *E. gallinarum*. Insects, including bees, termites, and beetles, host diverse species such as *E. mundtii*, *E. casseliflavus*, and *E. termites*, underscoring the ecological versatility of the genus [22,23]. An insect model study using *E. mundtii* isolated from flour moths showed improved survival rates in beetles challenged with *Bacillus thuringiensis*, demonstrating probiotic effects and suggesting its potential as a probiotic or therapeutic agent [27]. Another recent study using silkworms identified *E. mundtii* as a beneficial intestinal commensal that colonizes and adheres to the gut, enhances carbohydrate breakdown, improves intestinal microbiome balance, protects the insect from invading pathogens, and modulates host immunity [28]. A metagenomic study on the edible insect, soybean hawkmoth, identified *E. casseliflavus* and *E. pernyi* as the dominant gut bacteria, with *E. casseliflavus* contributing to nutrient metabolism and overall gut health [29].

Although some enterococci inhabit the gastrointestinal tract, their colonization of the nasal cavity in humans and animals is increasingly documented [30,31,32]. Studies have shown that the most common enterococci found in nasal samples are *E. faecium*, *E. faecalis*, and *E. casseliflavus* [32,33,34]. Nasal colonization by some enterococci may occur in hospitalized patients, immunosuppressed individuals, and patients with chronic rhinosinusitis or nasal devices [32]. Although they rarely cause infections on their own within the nasal sites, they may contribute to biofilm formation and sinonasal infections, or act as reservoirs for the acquisition and dissemination of antimicrobial resistance genes [34].

Some enterococci play a significant role in the gut ecosystem, contributing to immunomodulation, gut health, and metabolic maintenance [35,36]. However, disturbances to the intestinal microbiome caused by indiscriminate drug use can promote the spread of drug-resistant strains by wiping out susceptible strains while surviving bacteria recolonize the gut and, through fecal discharge, are introduced into the surrounding environment, further accentuating antimicrobial resistance [25,37]. Despite these risks, numerous studies have documented the benefits of some strains of *Enterococcus* spp. in the gut. For example, *E. casseliflavus* has exhibited considerable therapeutic promise, with clinical evidence indicating improved survival outcomes in hematopoietic stem cell transplant (HSCT) recipients [38]. Nevertheless, some *Enterococcus* spp. can also transform into opportunistic pathogens outside the gastrointestinal region, causing conditions such as heart infections, bloodstream infections, urinary tract infections, and systemic infections [39]. Among the diverse *Enterococcus* species shown in Figure 1, *E. faecium* and *E. faecalis* predominate within the gut and play imperative roles in gastrointestinal health maintenance and immune modulation [35,40]. Research confirms that introducing probiotic bacteria into the gut helps maintain microbial balance, supports digestion, and strengthens immune responses [41]. Specific *Enterococcus* strains have been identified as possessing notable probiotic and health-promoting properties through multiple mechanisms [42,43]. A well-documented mechanism is competitive exclusion, where *Enterococcus* strains compete with pathogens for binding sites on the intestinal wall, thereby blocking colonization by harmful bacteria [44].

Moreover, some enterococci synthesize bacteriocins (especially enterocins), antimicrobial compounds that inhibit the establishment of detrimental bacteria in the gastrointestinal tract, thereby contributing to gut microbiota stability and overall well-being [45,46]. Additionally, some *Enterococcus* spp. produce fatty acid metabolites, including short-chain fatty acids (SCFAs), which are critical mediators of intestinal homeostasis. These SCFAs serve as fuel for intestinal epithelial cells, enhance intestinal barrier integrity by upregulating tight junction protein expression, attenuate pro-inflammatory cascades, and participate in immunomodulatory processes [47]. Research indicated that including probiotic *E. faecalis* in juvenile crayfish diets can enhance growth, strengthen the immune system, and improve survival, making it a promising supplement for aquaculture [45]. Similarly, in dogs with chronic digestive issues, *E. faecalis* supplementation helps balance gut bacteria, leading to improved digestion and overall health [48].

**Figure 2:**
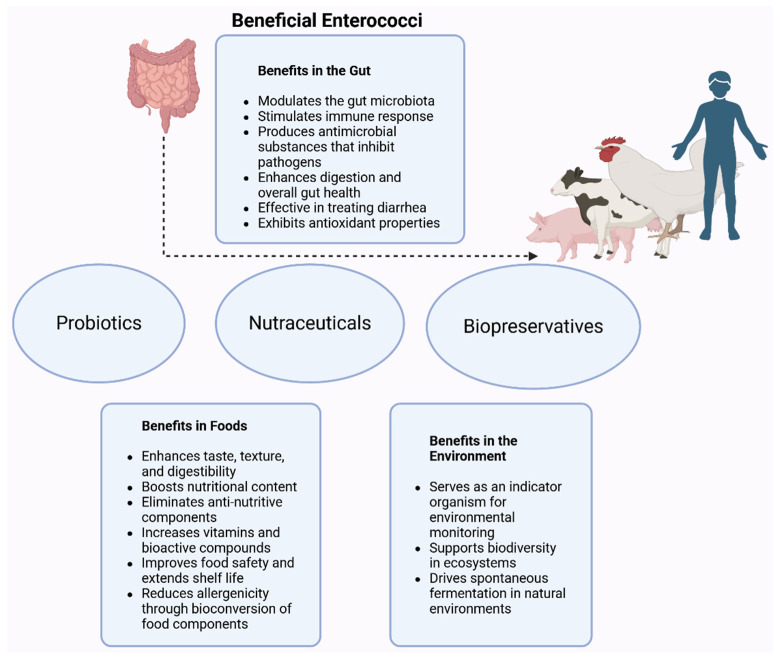
Benefits of *Enterococcus* spp. in the gastrointestinal tract of animals and humans, foods, and the environment.

Furthermore, some strains of *Enterococcus* spp. also contribute significantly to the gut microbiota by metabolizing polysaccharides, particularly indigestible dietary fibers, and converting them into bioavailable nutrients. Research by Basu et al. [49] demonstrated that supplementing dairy calves with *E. faecium* 669 significantly enhances body weight, average daily gain, and gastrointestinal health during the late preweaning phase. Notably, treated calves showed reduced incidence of diarrhea and lower rectal temperatures, suggesting enhanced gut barrier function and immune modulation. As illustrated in Figure 2, strains such as *E. faecium* and *E. faecalis* have shown promise as probiotic supplements, with demonstrated benefits for digestive efficiency, immune response, and growth performance.

### 2.2. The Environment

Enterococci enter and thrive in various environmental niches, primarily due to the discharge of fecal matter from humans, animals, and sewage, as well as agricultural runoff [50]. Their remarkable adaptability allows them to be widely distributed across diverse ecological habitats, including soils, plants, and water bodies [23,50,51,52,53,54]. This ability to persist and grow in harsh environments underscores their ecological importance and functional versatility. However, these intrinsic biological properties also make *Enterococcus* spp. particularly effective disseminators of drug resistance in the environment [51,52].

Common *Enterococcus* species found in soils, including agricultural, contaminated, and natural soils, are *E. hirae* and *E. faecium* [53,54]. *E. faecalis* and *E. faecium* are frequently detected in rivers and streams, often originating from soil runoff, wastewater, or fecal contamination [55]. Additionally, *E. faecalis*, *E. faecium*, and *E. casseliflavus* are commonly found in plants as well as in untreated and treated wastewater [51]. Within soil environments, some *Enterococcus* spp. persist as minor but significant components of the microbiota, contributing to elevated bacterial counts [23].

Beyond their ecological roles (Figure 2), certain *Enterococcus* spp. have valuable applications in environmental remediation [56,57]. For example, *Enterococcus* spp. designated Cdq4–2 contribute significantly to bioremediation when used alongside plants such as *Lolium perenne*, enhancing plant growth and facilitating heavy metal remediation [58]. Moreover, auxin-producing *Enterococcus* strains have demonstrated plant growth-promoting capabilities and the potential to produce nanobiofertilizers for sustainable agriculture and food security [59].

Despite these ecological benefits, some *Enterococcus* spp. pose significant challenges due to their pivotal role in propagating antibiotic resistance through environmental pathways. They are well-known reservoirs of resistance, driven by both intrinsic and acquired mechanisms [60]. Soil, polar lakes, and aquatic ecosystems, especially through runoff and wastewater discharge, serve as conduits for the spread of antibiotic-resistant *Enterococcus* strains, thereby contributing to public health risks [52]. For example, Rehman and colleagues [61] reported that bacterial strains from drinking water and sewer overflow carried harmful virulence genes and transferable plasmids, highlighting potential health threats. This research indicates that environmental exposure to some enterococci may increase the risk of antibiotic resistance transmission to humans. Common species such as *E. faecalis*, *E. faecium*, *E. hirae*, and *E. casseliflavus* are often found in these environments, and their highly adaptable genomes enable survival under harsh conditions, making them persistent concerns [51].

Although some *Enterococcus* strains are known to cause infections, especially in hospital settings, these bacteria also play valuable roles in nature and industry. For instance, they are key players in traditional fermented foods, where they initiate natural fermentation processes [62]. In many developing regions, where commercial starter cultures are not always accessible, wild *Enterococcus* strains from the environment serve as natural fermenters. This not only enables fermentation but also improves the taste, texture, and overall quality of foods such as fermented vegetables, grains, and dairy products [63,64].

Ademola et al. reported that the genus *Enterococcus* is pivotal in the natural fermentation of Nigerian traditional condiments, such as *Iru* and *Ogiri* [65]. They identified *Enterococcus* among the naturally occurring genera in the preparation environment of these condiments, with the potential to serve as starter cultures. Their involvement influences the taste, aroma, and nutrient content of the final products [66].

In addition to their relevance in spontaneous traditional food fermentation, *Enterococcus* spp. serve as fundamental components of ecosystem dynamics. Research indicates that these microorganisms actively shape microbial communities by participating in biogeochemical processes, particularly the mineralization of nitrogenous compounds and carbon-rich substrates in terrestrial and aquatic systems [60]. Their metabolic activities can promote detrital decomposition and maintain critical nutrient turnover rates, making them keystone taxa that contribute to environmental sustainability.

Furthermore, *Enterococcus* spp. serve as critical sentinels for tracking the flow of antimicrobial resistance (AMR) genes in ecosystems. Due to their remarkable genetic adaptability and capacity for horizontal gene transfer, these bacteria function as effective biomarkers for assessing AMR dissemination, particularly in wastewater and agriculturally impacted habitats [10]. The persistent detection of AMR in such environments highlights patterns of resistance spread, underscoring the need for deeper investigation into how natural systems contribute to AMR evolution and propagation.

### 2.3. Foods

Food is a complex ecosystem that provides an ideal habitat for most microorganisms. Enterococci are among the predominant microbes in fermented food products [17]. While *E. faecium* and *E. faecalis* are the most common species found in foods, *E. durans*, *E. hirae*, *E. lactis*, *E. olivae*, *E. italicus*, *E. gallinarum*, and *E. casseliflavus* are less predominant [13,67,68]. Several of these lesser-known enterococcal species have been isolated from various fermented and raw food sources. For example, *E. olivae* was isolated from Spanish fermented green olives [69], *E. camelliae* from fermented tea leaves in Thailand [70], *E. lactis* from milk and dairy products [71], and *E. thailandicus* from fermented sausage. Additionally, species such as *E. hirae*, *E. casseliflavus*, and *E. gallinarum* have been reported in raw meat samples from Ghana [60].

*Enterococcus* spp. play significant roles in various fermented foods (Figure 2), contributing to flavor development, food safety, and quality. These bacteria dominate cheeses, where their proteolytic and lipolytic activities produce volatile compounds that increase flavor complexity [72]. Strains such as *E. durans* and *E. hirae* have been notable in traditional cheeses, enhancing microbiota diversity [73]. Similarly, in dry-cured sausages and fermented meats, *E. faecium* and *E. faecalis* improve flavor and texture through enzymatic activities [74]. However, some dairy-derived and other fermented foods containing *Enterococcus* spp., particularly *E. faecium* and *E. faecalis*, have been recognized as potential reservoirs of virulence and antibiotic resistance genes [75]. For instance, a survey of Turkish cheese samples revealed isolates with high resistance to lincomycin, kanamycin, low-level gentamicin, rifampin, and tetracycline, alongside several resistance genes (*tetM*, *tetL*, *ermB*, *cat*, *aph(3′)-IIIa*, *ant(6)-Ia*, *aac(6′)-Ie-aph(2″)-Ia*) [76]. Similar patterns have been observed in other countries. In Ethiopia, a study of fermented foods, including probiotic yogurt sold in Addis Ababa, detected *Enterococcus* spp. in most samples, with many isolates carrying multiple virulence genes (*gelE*, *cylA/B/M*, *asa*, *efaA*, *cpd*, *ccf*, *cob*) and resistance markers for vancomycin (*vanC1*, *vanC2/C3*) and erythromycin (*ermB*, *ermC*), underscoring the need for stricter microbial quality control [63]. While *Enterococcus* strains from cheeses were found to harbor resistance determinants for vancomycin, tetracycline, and erythromycin in Portugal [77], fermented foods-associated enterococci from Nigeria displayed multidrug resistance and carried multiple AMR genes, including *parC*, *aac-li*, *ermB/ermC*, and *tetM* [78]. Likewise, a more recent study in Greece identified *Enterococcus* strains from raw sheep milk exhibiting resistance to tetracyclines, macrolides, and chloramphenicol, with some phenotypic resistance not explained by detectable resistance genes [79]. Collectively, these findings highlight the widespread occurrence of virulence factors and antimicrobial resistance determinants in *Enterococcus* from diverse fermented food sources, raising concerns about their potential role in the transmission of resistance through the food chain.

Unlike *E. faecalis* and *E. faecium*, non-dominant *Enterococcus* species generally harbor a narrower range of virulence and antibiotic resistance genes [80,81]. As a result, they are increasingly being explored and thoroughly assessed for probiotic applications, particularly given *E. faecium*’s association with nosocomial infections and its extensive repertoire of virulence and resistance determinants. One such species, *E. lactis*, is closely related to *E. faecium* but differs in carbohydrate metabolism and 16S rRNA sequence, supporting its classification as a distinct species [82,83]. *E. lactis* has been reported to be relatively safe for use in the food and health sectors. For instance, its strains harbor significantly fewer acquired resistance and virulence factors compared to *E. faecium* [71]. However, they carry intrinsic resistance genes such as *eatA* (T450I), *msr(C)*, and *aac(6′)-Ii*. They also express intrinsic virulence markers, including *ccpA*, *fnm*, and *lysM4*; however, their overall lower risk profile supports their potential as probiotics [84]. A comparative genomic study of isolates from dairy products, rice wine Koji, and human feces further confirmed the limited antibiotic resistance of *E. lactis* relative to *E. faecium*, and identified novel genetic targets for future resistance research [43].

Extending beyond food-associated contexts, Ye et al. recently identified *E. lactis* strain GL3 from honeybee larvae as a promising probiotic for enhancing honeybee health and controlling American foulbrood, a lethal larval disease caused by *Paenibacillus larvae* [85]. Strain GL3 demonstrated strong acid and bile tolerance, antioxidant activity, and potent inhibition of *P. larvae* [85]. Genomic analysis revealed traits for gut adaptation and the ability to produce the antibacterial compound aborycin, which could also serve as a therapeutic agent. Another species, *E. italicus* strain GGN10, lacked pathogenicity islands and tested negative for common resistance genes, including those for vancomycin, β-lactams, and gentamicin [86]. Similarly, *E. italicus* FM5 was non-hemolytic, displayed antioxidant activity, exhibited fermentation-enhancing traits, and was sensitive to a broad range of antibiotics, highlighting its safety for food applications and potential as a probiotic [81].

In fermented plant-based products like olives, sauerkraut, and kimchi, some beneficial enterococci often act as secondary microbiota, influencing fermentation dynamics without being essential to the process [87]. *E. faecium* and *E. faecalis* are prevalent in African fermented cereals, including *Ogi* (Nigeria), *Mawe* (Benin), and *Kenkey* (Ghana), where they contribute to acidification and microbial stability by inhibiting spoilage organisms and pathogens [66,88,89]. In legume-based condiments such as *dawadawa/Iru* (fermented African locust beans) and *Ogiri* (fermented melon seeds), *E. faecium* and *E. durans* enhance flavor through proteolysis and amino acid production, ensuring rapid fermentation and reducing spoilage risk [66]. These species are also prevalent in traditional dairy products, such as *Nunu* (West Africa), *Kule naoto*, and *Mursik* (East Africa), where they contribute to flavor development and inhibit pathogens through bacteriocin production [12,90]. While less prominent in alcoholic beverages, some enterococci may appear transiently in products such as palm wine, influencing early fermentation stages before being overtaken by yeasts and other bacteria [91,92]. Specific food-associated strains of *E. faecium* and *E. faecalis* are recognized for their probiotic potential, including their ability to modulate the gut microbiota, enhance immune responses, and compete with pathogens. For example, dietary supplementation with *E. faecium* has been shown to reduce diarrhea severity in weaned piglets, increase growth, and boost the abundance of beneficial gut microbes [93]. Similarly, *E. faecalis* strains have been associated with improved intestinal microbial balance and immunological homeostasis [94].

Despite their beneficial roles, some enterococci are notorious for both intrinsic and acquired antibiotic resistance, including resistance to vancomycin mediated by the *vanA* and *vanB* genes [68]. They act as reservoirs of resistance genes, which can be transferred to other foodborne spoilage and pathogenic bacteria, including *Staphylococcus aureus*. Additionally, some enterococci harbor virulence determinants such as aggregation substances, gelatinases, and cytolysins, making them potential opportunistic pathogens [95]. Their natural ability to transmit genetic material via mobile elements, including plasmids and transposons, facilitates the dissemination of resistance and virulence genes within microbial communities [96]. Therefore, the safety of food-associated *Enterococcus* strains varies significantly, with pathogenic potential largely dependent on the presence of virulence determinants. This dichotomy is exemplified by contrasting findings: while most Slovak Lump Cheese contains an *E. durans* strain devoid of virulence genes, indicating probiotic suitability [97], other dairy products harbor resistant, virulent *E. durans* strains [98]. Genomic studies corroborate this disparity, revealing that clinical isolates typically carry higher virulence gene loads than food-derived strains [99]. Such evidence highlights the importance of conducting strain-specific risk assessments prior to food applications.

Some strains of *Enterococcus* spp. significantly influence the organoleptic characteristics of fermented products through multiple metabolic pathways, including pH reduction via acid generation, protein degradation, fat breakdown, and citrate utilization [17]. While most *Enterococcus* isolates display limited acid production, *E. faecalis* strains demonstrate notably greater acidifying potential than *E. faecium* strains. Proteolytic activity appears limited in approximately 70% of strains examined, with *E. faecalis* consistently exhibiting the most pronounced protein-degrading capacity within the genus [100,101]. The *gelE* gene in *Enterococcus* is responsible for the degradation of casein, enhancing texture and aroma in fermented foods, especially dairy products. However, in hospital-associated strains, *gelE* is also linked to virulence, particularly in *E. faecalis* [102]. The pathogenic potential of *Enterococcus* depends on factors such as gene expression, environmental conditions, and strain-specific characteristics [98,103]. This highlights the importance of thorough genomic and phenotypic analyses when evaluating *Enterococcus* strains for food applications. In addition to protein degradation, *E. faecalis* demonstrates efficient citrate utilization, resulting in increased diacetyl synthesis—a crucial compound responsible for the characteristic creamy fragrance in fermented dairy products. This pronounced ability to generate flavor compounds distinguishes *E. faecalis* from *E. faecium*, which exhibits substantially reduced citrate metabolic activity [104,105].

Current shifts toward biologically derived food preservation methods have highlighted some *Enterococcus* spp. as potential biopreservatives. These microorganisms synthesize diverse antimicrobial metabolites, particularly enterocins and organic acids, which significantly suppress the growth of both pathogenic and spoilage organisms in food systems. A compelling example includes recent findings on *E. faecium* Smr18, whose enterocin ESmr18 exhibited potent anti-*Salmonella* activity in experimentally contaminated poultry matrices [106]. The safety profile of *E. faecium* Smr18 enhances its biopreservative potential, as evidenced by its antibiotic susceptibility to penicillin, ampicillin, vancomycin, and erythromycin and the absence of hemolytic activity, substantiating its suitability for food safety applications [107].

## 3. Multifaceted Beneficial Enterococci

### 3.1. Enterococcus Probiotics

The provenance of probiotics can be traced back to 2000 BC, when ancient civilizations began developing methods to preserve milk for extended periods. Through trial and error, they discovered how to produce fermented beverages by carefully observing changes in stored food [108]. These early societies noted that when fruits and grains were left in sealed containers, they transformed into wine and beer. While the mechanisms behind these transformations were not understood at the time, these practices laid the foundation for fermentation science [109].

A significant milestone in understanding fermentation occurred in 1856, when Louis Pasteur was consulted by a beetroot stillman whose production process yielded a sour, milk-like substance instead of alcohol. Pasteur analyzed the chemical composition of the problematic product and identified lactic acid as its primary byproduct. Using microscopy, he compared samples from containers that had undergone alcoholic fermentation with those producing lactic acid. His observations revealed distinct microbial populations: yeast cells were linked to alcoholic fermentation, while smaller bacterial cells were responsible for lactic acid fermentation [109]. This groundbreaking discovery not only established the distinction between alcoholic and lactic acid fermentation but also highlighted the role of microorganisms in these processes.

Building on Pasteur’s discoveries, the early 1900s experienced a surge in advances in the application of lactic acid bacteria as probiotics. A landmark development occurred in 1935, when Dr. Minoru Shirota introduced Yakult, a fermented dairy product containing *Lactobacillus casei* Shirota. As a product that enhances intestinal health and promotes longevity, Yakult marked the beginning of the commercial era of probiotics. Its success has spurred global interest in developing nutraceuticals to support gastrointestinal health and overall well-being [110].

The probiotic potential of some strains of *Enterococcus* spp. began to garner attention in the mid-20th century. Systematic studies have revealed their ability to withstand harsh gastrointestinal conditions and colonize the gut, making them robust candidates for probiotic applications. *E. faecalis* and *E. faecium* were identified by researchers as strains with significant health benefits (Table 1), laying the groundwork for their inclusion in probiotic formulations [111].

In the 1980s and 1990s, some *Enterococcus* strains gained prominence, particularly in Europe, where they were incorporated into commercial probiotic products [108]. These bacteria possess a range of beneficial properties, including the production of antimicrobial peptides, such as enterocin A and B, modulation of the gut microbiome, and the inhibition of harmful pathogens. Therefore, some beneficial *Enterococcus* strains are effective in treating various gastrointestinal diseases, such as diarrhea and irritable bowel disorder [16].

Several commercial products containing *Enterococcus* strains are available globally and are useful for both human and animal health. One notable product is Symbioflor 1, which contains *E. faecalis*. Manufactured by SymbioPharm GmbH in Germany, this probiotic is specifically used to treat recurrent upper respiratory tract infections, highlighting its therapeutic potential beyond gastrointestinal health [112]. Another product, SF68^®^, features *E. faecium* SF68^®^ (NCIMB 10415), a strain extensively utilized for supplementation in animal feeds and as a medicine for humans. Produced by Cerbios-Pharma SA in Switzerland, SF68^®^ has gained popularity for its effectiveness in enhancing digestive health and host immune function [113]. In the field of animal health, Cylactin^®^, also manufactured by Cerbios-Pharma SA, uses *E. faecium* as a feed supplement to improve growth and feed efficiency in livestock; this product is particularly prevalent in Switzerland and has demonstrated benefits in chicken and pig farming [114]. Similarly, Medilac-Vita, a product containing *E. faecium*, is widely used in China for the treatment of infantile enteritis, demonstrating its application in pediatric care [115]. Other notable *Enterococcus* strains include *E. lactis* DSM 7134, *E. lactis* NCIMB 10415, and *E. faecium* 669. These strains are integrated into various probiotic formulations to treat some health-related issues [17,18,116]. Despite their widespread use, *Enterococcus* probiotics are subject to stringent regulatory and safety evaluations. In the European Union, regulations such as 700/524/EEC and EG 1831/2003 permit the utilization of beneficial strains of *Enterococcus* spp. in animal feed [112], whereas the United States allows selected *Enterococcus* strains in its list of direct-fed microorganisms for livestock. However, the genus does not currently hold Generally Recognized as Safe (GRAS) status nor is it included in the Qualified Presumption of Safety (QPS) list, reflecting ongoing scrutiny of its safety profile [5]. Regardless, the probiotic potential of some enterococci is significantly supported by studies and the literature, which highlight their diverse benefits [111,117].

Probiotic qualification requires microorganisms to meet stringent physiological and genetic standards, including gastric and biliary tract survival competence, epithelial adhesion capacity, host safety assurance, antimicrobial compound production, and the absence of mobile genetic elements encoding virulence or resistance factors [112]. Some strains of *Enterococcus* spp. naturally satisfy these requirements through their gastrointestinal adaptation, positioning them as compelling probiotic candidates. While selected strains, such as *E. faecium* LCW 44 and *E. durans* 6HL, have broad-spectrum antibacterial efficacy against both Gram-positive and Gram-negative pathogens, only a limited number have achieved regulatory approval for commercial probiotic use [118]. Research has identified several *Enterococcus* strains with distinct probiotic attributes (Table 1). For example, *E. durans* M4-5 exhibits significant intestinal health benefits due to its potent anti-inflammatory effects on gut epithelial tissue [119]. Another noteworthy example, *E. mundtii* ST4SA, produces broad-spectrum antimicrobial compounds that are effective against multiple pathogenic species, further expanding the probiotic potential of this genus [120]. 

Emerging research highlights several *Enterococcus* spp. strains as producers of bioactive peptides with cardiovascular benefits. These microbial metabolites exert antihypertensive effects by selectively inhibiting angiotensin-converting enzyme (ACE), the key regulator of the renin-angiotensin system [121,122]. By blocking the conversion of angiotensin I to its potent vasoconstrictor form (angiotensin II), these peptides promote vascular relaxation and enhance renal sodium elimination, collectively reducing blood pressure [123]. With hypertension affecting nearly one-third of adults worldwide [124], further investigation into *Enterococcus*-derived ACE inhibitors represents a critical avenue for developing novel therapeutic interventions. Another health benefit of some beneficial enterococci is demonstrated in a stem cell transplantation study, where *E. casseliflavus* exhibited considerable therapeutic promise, with clinical evidence indicating improved survival outcomes [38]. Furthermore, another species, *E. gallinarum*, has been shown to possess significant antitumor effects, highlighting its therapeutic applications in oncology [125].

Multiple enterococci strains have demonstrated significant potential as functional supplements in animal husbandry applications. Studies indicate that these microorganisms can be effectively used in diverse agricultural roles, including livestock feed enrichment, silage fermentation enhancement, gastrointestinal microbiota modulation, and antibiotic-alternative therapies (Table 1). Research has demonstrated the multifaceted benefits of *E. faecium* supplementation across poultry and aquaculture systems. An earlier investigation revealed that *E. faecium* enhances broiler growth performance, promotes intestinal villus development, and modulates caecal microbiota composition during *E. coli* K88 infection [126]. Subsequent studies confirmed these findings, with Huang et al. reporting improved survival rates, enhanced immune responses, and maintenance of gut barrier function in broiler chickens challenged with *E. coli* O78 [127]. The probiotic effects extend to aquatic species, where 14 days of *E. faecium* supplementation in Tilapia feed significantly improved growth parameters and immune competence, while prolonged administration conferred protection against *Aeromonas hydrophila* infection [128].

Additionally, documented benefits span companion animals, aquaculture species, and other production livestock, where supplementation with some strains of *Enterococcus* spp. has been associated with enhanced growth performance, pathogen inhibition, immune system potentiation, improved metabolic efficiency, reduced diarrhoeal incidence, maintenance of intestinal barrier function, and anti-inflammatory effects [85,105,113,129,130,131,132].

**Table 1:** Applications of potential probiotic *Enterococcus* spp.

Probiotic Organism	Component	Model of Study	Benefits	Reference
**Animal Health Application**				
***E. faecium***	Viable cells	Broiler chickens	Promotes growth performance, improves intestinal morphology, and beneficially manipulates the cecal microflora in broilers challenged with *E. coli* K88.	[126]
***E. faecium* NCIMB 10415**	Viable cells	Broiler chickens	Improves growth performance, meat quality, and antioxidant capacity of muscle	[131]
***E. facium***	Viable cells	Mice	Antagonized *Salmonella* Typhimurium alleviating inflammatory injury through the NF-κB/NLRP3/IL-1β signaling pathway.	[133]
***E. faecium* R8a**	Viable cells	Giant tiger shrimps (*Penaeus monodon*)	It enhances immune response, combats intestinal inflammation, repairs gut barrier dysfunction, and rebalances gut flora.	[134]
***E. faecium***	Viable cells	Laying hens	Antimicrobial activity against *Salmonella* Enteritidis, modulation of the gut microbiome, and enhances overall health and laying capacity.	[135]
***E. faecium***	Viable cells	Big-belly seahorses (*Hippocampus abdominalis*)	Improves growth, intestinal health, and immunity. Increase in survival rate by 50% against pathogenic *Edwardsiella tarda* challenge	[136]
***E. faecium***	Viable cells	Nile Tilapia (*Oreochromis niloticus*)	Enhances immunity and improves intestinal flora composition. Resistance against Francisellosis and Streptococcosis	[137]
***E. faecium* NCIMB11181**	Viable cells	Broiler chickens	Inhibits *E. coli* O78 proliferation, improves intestinal morphology, and enhances immune response	[127]
***E. faecium* EGY_NRC1**	Viable cells	Lactating Holstein cows	Increased total tract digestibility of dry matter, neutral and acid detergent fiber, daily milk production, and feed efficiency. Improved organic matter, crude protein, and nonstructural carbohydrates digestibility. Increased serum glucose and decreased serum cholesterol	[138]
***E. faecium***	Viable cells	Milk-producing Holsteins	Improves animal growth performance and intestinal health	[138]
***E. faecium***	Heat-killed cells	Male minks	Enhances growth performance, digestibility, immune function, and intestinal development.	[139]
***E. durans***	Viable cells	Broiler chickens	Maintains gut epithelial health and exhibits antagonistic effects against pathogens, and improves animal weight	[140]
**Bio-therapeutic Application**				
***E. faecium* SF68**	Viable cells	Mice	Adjusted the composition of the dysbiotic microbiota of High Fat Diet-fed animals, thus ameliorating clinical conditions and exerting anti-obesity effects.	[141]
***E. faecium* KU22001**	Heat-killed cells	In vitro	Antioxidative and immunity-enhancing effects	[132]
***E. lactis***	Viable cells	In vitro	Cholesterol-lowering effects	[142]
***E. faecuim***	Viable cells	In vitro	Cholesterol-lowering activity	[143]
***E. faecalis* CECT 5728**	Viable cells	Murine	Antihypertensive activity	[144]
***E. faecalis* EF-2001**	Heat-killed cells	Mice	Induces human dermal papilla cell proliferation and hair regrowth	[145]
***E. faecalis* EF-2001**	Heat-killed cells	Mice	Activates AMPK signaling in the liver, which attenuates lipid accumulation in diet-induced obese mice	[146]
***E. faecalis* KH2**	Heat-killed cells	Mice	Promotes re-epithelialization and granulation tissue formation during skin wound-healing	[147]
***Enterococcus* spp.**	Viable cells	Patients with liver cirrhosis	Improves the hepatic function of patients with liver cirrhosis. Improves haematobiochemical parameters and gut microbial composition	[148]
***E. faecium* 96B4**	Viable cells	In vitro	Antilisterial activity and fatty acids biotransformation	[149]
***E. faecium* FUA027**	Viable cells	In vitro	Survival in simulated gastrointestinal environments, antimicrobial and antioxidant activity	[150]
**Bacterial Infection Application**				
***E. faecium* TM39**	Heat-killed cells	Mice	Protection against *Salmonella* infection. Enhanced immune response in *Salmonella* infection	[151]
***E. faecium* 9 N-2**	Viable cells	In vitro	Antibacterial activity against common foodborne pathogens, survivability in gut conditions	[152]
***E. faecium* DC-K7 and DC-K9**	Viable cells	Mice	Promote a healthy balance of gut microbiota following antibiotic treatment. Suppresses the growth of gut pathogens	[153]
**Chemotherapeutic Application**				
***E. hirae***	Viable cells	Mice	Anticancer properties. Promote signaling of the innate immune sensor protein NOD2 and improved immunotherapy responses	[154]
***E. faecium* com15, *E. faecalis***	Viable cells	Mice	Enhanced cognate anticancer immune responses. Improves immune response to checkpoint inhibitor immunotherapy	[155]
***E. hirae***	Viable cells	Mice	Enhances anti-tumor immune responses during cyclophosphamide (CTX) therapy	[156]
**Anti-fungal Application**				
***E. mundtii* CRL35 and *E. faecium* ST88Ch**	Viable cells	In vitro	Anti-*Candida albicans* activity	[157]
**Food Application**				
***E. durans* OP268118**	Viable cells	In vitro	Functional microbial cultures for bio-preservation and fermentation of dairy matrices	[158]
***E. faecium* UBEF-41**	Viable cells	Artisanal dry-fermented sausages	Inhibits the growth of undesirable microorganisms and exhibits preservative effects in sausage products	[159]
***E. faecium* (ATCC 8459)**	Viable cells	Fermented dry-cured sausage	Optimizes the sensory profile of the sausage	[160]

### 3.2. Enterococcus Parabiotics

Parabiotics represent a novel class of nonviable microbial preparations that retain biological activity despite cellular inactivation [161]. These inactivated probiotic derivatives maintain immunomodulatory properties and can be produced through various inactivation techniques, including thermal treatment, ultraviolet irradiation, chemical exposure, and ultrasonic disruption [162]. The stability of parabiotics under processing conditions makes them particularly valuable for functional food applications, as they can deliver health benefits even after exposure to manufacturing processes that would compromise the viability of live probiotics [163]. However, unlike their viable counterparts, parabiotics lack the capacity for in situ production of biologically active compounds, such as antimicrobial peptides, during gastrointestinal transit, resulting in a more constrained spectrum of functional effects [164].

Emerging research indicates that inactivated *Enterococcus* strains retain significant biological activity. Fan et al. reported that heat-treated *E. faecalis* EF-2001 effectively attenuated adipose tissue deposition in obese rodent models [146]. Complementary findings demonstrate that thermally inactivated lactic acid bacteria consortia containing *E. faecium* TM39 not only prevent *Salmonella* establishment in murine hosts but also stimulate protective immune responses [151]. Similarly, Lee et al. found that heat-inactivated *E. faecalis* reduced liver fat and prevented damage in hamsters fed a high-fat diet. These findings highlight the promising potential of parabiotics for future therapeutic and functional applications [165].

Heat-killed *Enterococcus* strains have also shown great promise in diverse therapeutic areas, including cancer treatment, hair regrowth, biofilm inhibition, and gut health. Notably, specific *E. faecium* isolates (KU22001, KU22002, KU22005) derived from infant fecal microbiota exhibited measurable antitumor activity by inhibiting malignant cell growth, as assessed through tetrazolium-based viability assays [164]. Among these, strain KU22001 was particularly effective at inducing apoptosis and cell cycle arrest, suggesting its potential as a cancer and cancer-associated therapeutic agent. In addition to anticancer effects, heat-killed *E. faecalis* EF-2001 has demonstrated remarkable efficacy in promoting hair growth and preventing hair loss. Studies have revealed that EF-2001 enhances the proliferation of cells within hair follicles and stimulates hair regrowth in experimental models, positioning it as a promising alternative or complement to conventional treatments, such as minoxidil [145]. This underscores its potential to address hair-related disorders while minimizing the side effects often associated with existing therapies.

Furthermore, heat-killed probiotics, including some *Enterococcus* strains, have been shown to inhibit biofilm formation and disperse established biofilms, offering a novel approach to managing urinary tract infections (UTIs). UTIs are often complicated by biofilm-forming uropathogens and antibiotic resistance, posing significant healthcare challenges. Research has demonstrated that heat-killed probiotic bacteria, including *Enterococcus*, can effectively disrupt biofilms formed by pathogens such as uropathogenic *Escherichia coli* (UPEC), *Klebsiella pneumoniae*, and methicillin-resistant strains [166]. This capability not only prevents biofilm establishment but also reduces bacterial loads, suggesting a role for parabiotics in preventing recurrent UTIs.

### 3.3. Enterococcus Bacteriocins

**Figure 3:**
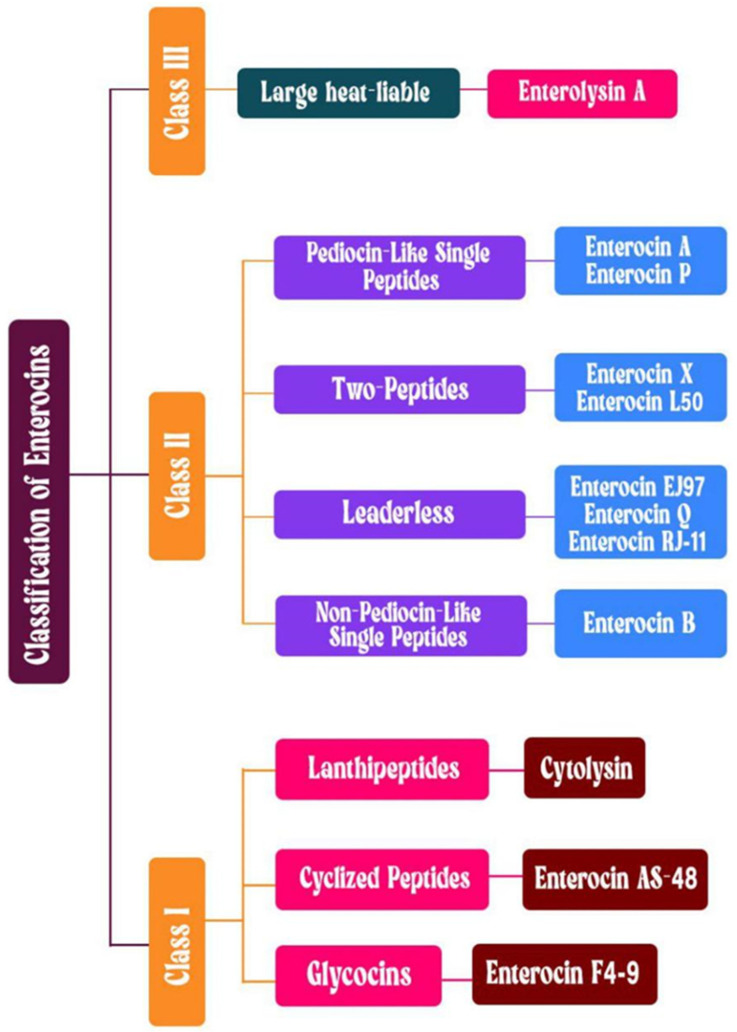
Classification of bacteriocins produced by enterococci (adapted and modified [170]).

Some *Enterococcus* strains produce antimicrobial peptides called bacteriocins, which are secreted extracellularly and inhibit the growth of various pathogenic bacteria. The classification of bacteriocins involves multiple biochemical parameters, including structural characteristics, size distribution, specific enzymatic alterations, and distinct genetic pathways [167,168]. Bacteriocins are currently categorized into three distinct classes according to the most recent classification system (Figure 3). Class I bacteriocins are characterized by their heat-stable peptide structure, which undergoes specific modifications after translation, resulting in their distinct properties, such as enterocin AS-48, enterocin F4-9, and cytolysin. Class II includes heat-stable, unmodified peptides, which form the largest group, including enterocin A, enterocin P, enterocin DD14, enterocin K1, enterocin SEK4, enterocin L50, enterocin B, bacteriocin 32 (Bac32), and EF478. Class III comprises large, heat-labile proteins, such as enterolysin A, and Bac41 [168,169].

Cytolysin, synthesized by *E. faecalis*, is a hemolytic virulence factor that contributes to human disease and increased mortality [171]. The cytolysin operon contains the genes *CylLL* and *CylLS*, which code for the two distinct subunits that comprise the cytolysin molecule. These subunits are essential for the toxic activity of cytolysin, which targets mammalian cells and Gram-positive bacteria [172].

Enterocin AS-48, a circular bacteriocin also synthesized by *E. faecalis*, features a unique head-to-tail circular structure composed of 70 amino acids arranged into five alpha-helices. This cationic peptide disrupts bacterial membranes, resulting in cell permeabilization and subsequent cell death [46]. This compound has been shown to exhibit potent antagonism against Gram-positive pathogens. Similarly, Enterocin AS-48 exhibits enhanced activity against Gram-negative bacteria when combined with outer membrane-permeabilizing agents [173]. Enterocin AS-48 is produced during both the exponential and stationary growth phases and can be obtained in high yields when whey-based substrates are used [168].

Enterocin F4-9 contains glycosylated residues that are important for its antimicrobial activity. The enterocin F4-9 biosynthesis-related gene cluster was cloned and expressed in *E. faecalis* JH2-2, resulting in a peptide with improved activity against bacteria [174]. The synthesis of enterocin F4-9 involves a two-step split of the leader peptide by the *EnfT* protease domain and extracellular proteases. Glycosylation, mediated by *enfC*, is critical for both secretion and activity, whereas *enfI* provides immunity to producing cells; this sheds light on the minimal gene set necessary for glycocin biosynthesis and reveals a novel mechanism of glycocin production [175].

Bacteriocins 31 (bac31) and 32 (bac32) have been isolated from *E. faecium* strains found in dairy products, such as traditional fresh soft cheese and raw cow’s milk. Both have demonstrated activity against important foodborne pathogens, including *L. monocytogenes* [176]. In contrast, Bac43 was originally isolated from vancomycin-resistant *Enterococcus* (VRE) clinical isolates in a hospital setting and has shown a dramatic increase in prevalence, being highly associated with emerging *E. faecium* MLSTs in clinical cohorts over time. Despite its clinical origin, Bac43 is also active against *L. monocytogenes* and various *Enterococcus* species [177]. Additionally, a bacteriocin called Pneumolancidin, initially characterized from *Streptococcus pneumoniae*, has been identified through homology in patient-derived *E. faecium* isolates in a recent study [178]. Most bacteriocins primarily cause cell lysis by targeting the cell membrane or sugar uptake systems. Their generally narrow spectrum of activity makes them advantageous for targeted treatment or biocontrol strategies, especially in combating the continuous emergence of multidrug-resistant pathogens [179].

Some enterococcal bacteriocins exhibit broad-spectrum antagonistic activity against clinically significant pathogens, including *Listeria monocytogenes*, *S. aureus*, *Escherichia coli*, and *Salmonella* spp. (Table 2). Their bactericidal mechanisms are diverse and target-specific, including membrane permeabilization and leakage of bacterial cellular content (characteristic of pediocin-like variants), inhibition of cell wall biosynthesis via lipid II binding (exhibited by lantibiotic-class enterocins), enzymatic degradation of peptidoglycan layers (demonstrated by large bacteriolysins such as enterolysin A), and disruption of essential cellular processes, including nucleic acid metabolism and translation machinery [180,181].

Bacteriocins have significant potential as natural preservatives in fermented food systems, particularly in dairy applications (Table 2). These antimicrobial peptides show synergistic activity when combined with other lactic acid bacteria, making them effective components in multi-strain fermentation cultures [121,182]. Specific variants, including enterocins A, B, and AS-48, display potent inhibitory effects against common foodborne pathogens such as *Salmonella enterica* and *E. coli*, effectively controlling their growth in food matrices. Furthermore, enterocins can be combined with bacteriophages for enhanced pathogen control. For example, Baños et al. demonstrated the use of enterocin AS-48 in combination with phage P100 to suppress the proliferation of *L. monocytogenes* in fish [183]. Various studies have shown the strong antimicrobial properties of enterocins against pathogens such as *Streptococcus pyogenes*, *S. suis*, *Clostridium perfringens*, *E. cecorum*, *E. faecalis*, *Pseudomonas aeruginosa*, *Campylobacter coli*, *Bacillus cereus*, and *S. aureus*, highlighting their potential for combating foodborne illnesses caused by these pathogens [184,185].

**Table 2:** Enterococci bacteriocins and their potential applications.

Bacteriocin	Producing *Enterococcus* Strain	Mode of Action	Model	Effects	Reference
**Microbiome modulation**					
**Enterocin (Ent7420)**	*E. faecium* CCM7420 (EF2019)	Stimulating the host’s immune reaction	Broiler rabbits	Immune-regulating effects. Antibacterial and anticoccidial effect. Improves serum biochemistry parameters, immunity, jejunal morphology, weight gains, feed conversion ratio, and meat quality	[186]
**Enterocin A/P**	*E. faecium* P13	Optimizes immune activity and gut flora composition	Rabbits	Suppression of gut pathogens combined with immune-modulatory influence	[169]
**Bio-preservation/food safety**					
**Enterocin AS-48**	*E. faecium* EK13 (CCM 7419)	Cytoplasmic membrane perturbation	Canned fruits and vegetables	Inhibit *Bacillus coagulans* in canned fruit and vegetable foods	[187]
**Enterocin AS-48**	*E. faecium*	Creation of nanopores in the lipid bilayer	Rice-Based Foods	Inhibition of Toxicogenic *Bacillus cereus*	[173]
**Enterocin F4-9**	*E. faecalis* F4-9	Suppression of bacterial growth	In vitro	Foodborne pathogens	[175]
**Enterocin AS-48**	*E. faecalis*	Pore formation in the cell membrane	Blueberries	Reduction in the viable counts of *Enterobacteriaceae*, *Salmonella*, and coliforms	[188]
**Enterocin-like** **substances**	*E. faecium* X2893 and X2906	Not included	In vitro	Antimicrobial activity against poultry-associated *Clostridium perfringens*	[189]
**Enterocin B**	*E. lactis* 4CP3	Disintegration of cellular envelope	In vitro	*C. perfringens*, *L. monocytogenes*, *Propionibacterium* spp., *C. sporogenes*, and *C. tyrobutyricum*	[168]
**Enterocin SEK4**	*E. faecalis* K-4	Pore formation in cell membrane of target organisms	In vitro	*C. perfringens*, *B. subtilis*, and *L. monocytogenes*	[168]
**Enterocin LD3**	*E. hirae* LD3	Cell membrane disruption	Fruit juice	Inhibition of *Salmonella enterica* subsp. *enterica* serovar Typhimurium ATCC 13311 in fruit juice	[190]
**Enterocin TJUQ1**	*E. faecium* TJUQ1	Disrupts cell membrane, leading to cell lysis	In vitro	Antimicrobial activity against *L. monocytogenes* CMCC 1595	[191]
**Enterocin CCM 4231**	*E. faecium* CCM 4231	Formation of pores in cell membrane	In vitro	*S. aureus* and *L. monocytogenes*	[192]
**Enterocin KT2W2G**	*E. faecalis* KT2W2G	Cell Membrane Depolarization	Banana peels	Inhibits the growth of spoilage microorganisms isolated from spoiled banana peel.	[193]
**Enterocin ABP**	*E. faecium* KE82	Not included	Cheese production plant	Enhances the inactivation of *L. monocytogenes* during the production of cheeses	[194]
**Enterocin DD14**	*E. faecalis* 14	Disrupts cell membranes and cell walls, causing cytoplasmic leakage and cell death.	In vitro and in vivo	Antimicrobial activity against *L. monocytogenes*, *C. perfringens*, *Enterococcus faecalis*, and methicillin-resistant *Staphylococcus aureus.*	[180]
**Enterocin P**	*Bioengineered Pichia pastoris*	Interacts with negatively charged bacterial membranes, causing cell death.	In vitro	Inhibition of Gram-positive pathogens, including *L. monocytogenes*, *S. aureus*, and *Clostridium* spp.	[195]
**Enterocin A**	*E. faecium* T136	Formation of pores in the cell membrane	In vitro	Anti-*L. monocytogenes* activity	[181]
**Enterocin HDX-2**	*E. faecium* HDX-2	Disrupts membrane integrity, leading to cell lysis.	In vitro	Inhibition of foodborne pathogens and spoilage bacteria.	[196]
**Enterocin L50**	*E. faecium* L50	Membrane disintegration leading to cell death	In vitro	Anti-*C. perfringens* activity	[190]
**Bacterial infections**					
**Enterocin A/P**	*E. faecium* EK13	Immunoglobulin A (IgA) production	Rabbits	Improves rabbits’ growth, immunity, and growth of rabbits, and therapeutic potential against staphylococcal infections	[169]
**Enterocin 416K1**	*E. casseliflavus* IM 416K1	Cell membrane disruption	In vitro	Antimicrobial activity against *L. monocytogenes*	[168]
**EntK1 and EntEJ97**	*E. faecium*	Decrease in cell membrane integrity	Mice	Treatment of systemic vancomycin-resistant enterococci infections	[197]
**Enterocin LD3**	*E. hirae* LD3	Cause efflux of intracellular ions and decrease in cell membrane integrity	In vitro	Antimicrobial activity against Gram-negative bacteria	[198]
**Enterocin OS13**	*E. faecalis* OS13	Formation of pores in the cell membrane	In vitro	Inhibitory activity against nosocomial enterococci	[199]
**Enterolysin A**	*E. durans* NT21	Cytoplasmic membrane perturbation	In vitro	Antimicrobial activity against *S. aureus*, *M. luteus*, *Salmonella* Typhi, and *E. faecalis*	[200]
**Enterocin E20C**	*E. hirae* 20C	Cell membrane disruption	In vitro	Antimicrobial activity against *S. enterica*	[201]
**Durancin 16A**	*E. durans* 16A	Membrane perforation	In vitro	Inhibition of *C. difficle*, *S. aureus*, and *Enterococcus* (vancomycin-resistant strains)	[167]
**Enterocin AS-48**	*E. faecalis*	Disintegration of cellular envelope	In vitro	Antimicrobial activity against *Mycobacterium tuberculosis*	[202]
**Antiviral**					
**Enterocin CRL 35**	*E. mundtii* CRL35	suppression of the late stages of replication	In vitro	Inhibition of Herpes Simplex Virus-1 and Herpes Simplex Virus-2	[203]
**Enterocin GEn17**	*E. durans*	Suppression of viral genome replication	In vitro	Inhibition of poliovirus (PV-1) and herpes simplex virus 1 (HSV-1)	[204]
**Enterocin AAR-71**	*E. faecalis* AAR-71	Suppression of viral genome replication	In vitro	Antiviral inhibitory activity and immune system stimulation	[205]
**Bacteriocin ST5Ha**	*E. faecium* ST5Ha	Inhibition of the key replication cycle	In vitro	Anti-herpes simplex virus type 1 (HSV-1) activity	[206]
**EntDD14**	*E. faecalis* 14	Disruption of virus assembly	In vitro	Antiviral activity against HSV	[180]
**Antifungal**					
**Enterocin UNAD 046**	*E. faecalis*	Loss of cell wall integrity	In vitro	Activity against *Aspergillus niger*, *Fusarium oxysporum*, *Pythium ultimum*, and *Penicillium expansum*	[207]
**Enterocin A, B, and P**	*E. lactis* 4CP3	Decreased mitochondrial membrane potential	In vitro	Antifungal activity against *A. niger* A79, *Bacillus thuringiensis, F. equiseti* F97	[208]
**Enterocin CHQS**	*E. faecalis* TG2	Peptidoglycan disruption with concurrent oxidative stress	In vitro	Antifungal activity against *Candida albicans*	[209]
**Enterocin Gr17**	*E. faecalis*	Loss of cell wall integrity	Salmon fillets	Antifungal activity, enhances sensory properties and shelf life of liquid-smoked salmon fillets	[210]
**Anti-cancer**					
**Enterocin 12A**	*E. faecium*	Permeabilization of the cancer cell plasma membrane	In vitro	Anticancer activity against human colon, gastric (HT-29, Caco-2, and AGS), cervical (HeLa) cancer cells	[211]
**Enterocin A**	*E. faecium (por1)*	Triggering late-stage cell death and cell cycle arrest	In vitro	Activity against gastric cancer cell lines	[212]
**Enterocin (LNS18)**	*E. thailandicus*	Cancer cell plasma membrane poration	In vitro	Activity against HepG2 cancer cells	[213]
**Enterocin P**	*E. faecium* P13	Attenuation of oncocytic membrane porosity	In vitro	Activity against cancer cell lines (SW1353, HUH7, Huh-7.5, C26, B16F0)	[214]
**Anti-protozoan**					
**Enterocin AS-48**	*E. faecalis* strain UGRA10	Selective mitochondrial targeting and the formation of highly reactive oxygen molecules (ROS).	In vitro	Activity against *Leishmania* spp.	[215]
**Enterocin AS-48**	*E. faecalis* UGRA10	Mitochondria depolarization	Mice	Effective for the treatment of Chagas’ disease	[216]
**Enterocin AS-48**	*E. faecalis* UGRA10	Affects and destroys cellular compartments	In vitro	Autophagic-related *Trypanosoma brucei* inhibition	[217]
**Durancin-like** **and Enterocin M**	*E. durans* ED26E/7	Formation of pores in cytoplasmic membrane	In vitro and mice	Amelioration of *Trichinella spiralis*-associated infections	[218]

Research has revealed distinct antimicrobial profiles among bacteriocin variants. The bacteriocin CRL35 exhibits potent listericidal activity as a standalone antimicrobial agent. In contrast, the engineered chimeric peptide Ent35-MccV displays broad-spectrum efficacy against resistant clinical strains, particularly *E. coli* isolates from healthcare settings [219,220]. Another significant antagonistic effect is observed when enterocin Durancin 61A is combined with reuterin against pathogens like *Clostridium difficile*, indicating its potential for treating gastrointestinal infections [167]. Emerging research also highlights the therapeutic potential of some enterocins against antibiotic-resistant pathogens. For example, durancin 61A demonstrates inhibitory activity against clinical MRSA isolates, exhibiting both standalone efficacy and synergistic effects with vancomycin therapy [167]. Complementary studies have identified enterocins DD28 and DD93 as promising candidates for treating MRSA infections, suggesting their potential application in antimicrobial strategies [221].

Contemporary research has focused on optimizing bacteriocins through chemical engineering to increase their pharmaceutical potential [129]. With this approach, Ross et al. developed novel antimicrobial peptides by structurally mimicking the α-helical domain of enterocin AS-48 [71]. Their engineered peptide library demonstrated potent antimicrobial efficacy across multiple bacterial strains, with certain variants exhibiting significant bactericidal activity at nanomolar concentrations while maintaining excellent human cell biocompatibility [222]. In light of the escalating antimicrobial resistance crisis, these findings position modified enterocins as promising candidates for next-generation therapeutics, warranting expanded investigation into their clinical applications (Table 2).

## 4. Safety Concerns Associated with the Use of *Enterococcus* spp.

### 4.1. Pathogenic Potential of Enterococcus spp.

**Figure 4:**
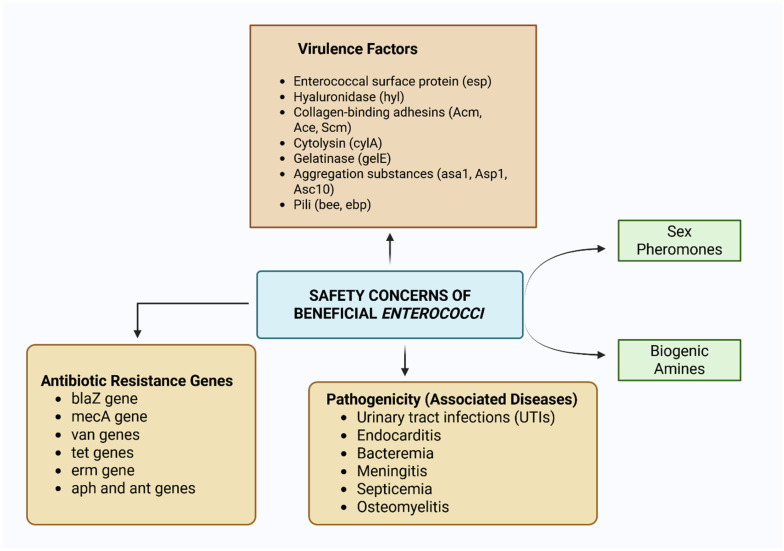
Major safety concerns associated with enterococci.

**Figure 5:**
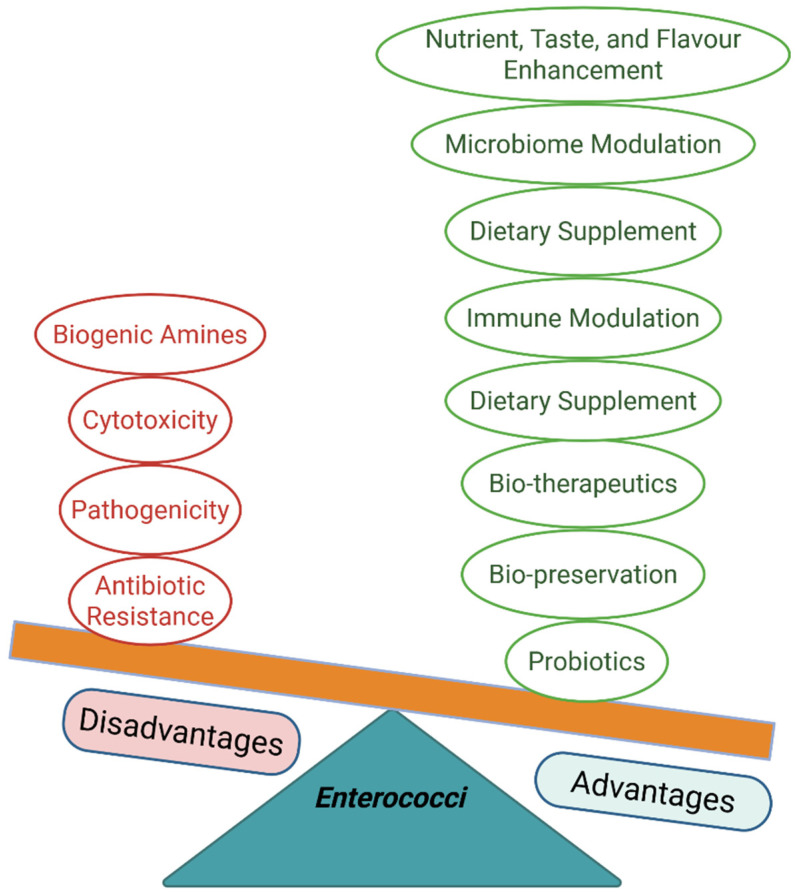
Highlights of the advantages and disadvantages of *Enterococcus* on a scale of impact.

Apart from the beneficial ecological roles played by different enterococci, some strains of *Enterococcus* spp. are widely recognized as clinically important pathogens, raising safety concerns about their use as probiotics (Figure 4 and Figure 5). Within healthcare settings, *E. faecalis* and *E. faecium* have become predominant nosocomial threats, with distinct pathogenic profiles. While *E. faecalis* demonstrates greater virulence potential, *E. faecium* shows a heightened propensity to develop multidrug resistance, particularly against glycopeptide antibiotics such as vancomycin [223,224].

Enterococcal infections primarily manifest in vulnerable patient populations, including immunodeficient individuals, critically ill patients, and those undergoing intensive medical interventions. Extended antimicrobial therapy and impaired host defenses represent key predisposing factors for nosocomial acquisition of drug-resistant enterococcal strains. These pathogens demonstrate remarkable clinical versatility and can induce diverse pathological states, ranging from catheter-associated urinary tract infections to life-threatening systemic conditions, such as endocardial infections, bloodstream infections, neonatal sepsis, infections following surgery or burns, and meningitis [224,225].

A global monitoring report from over 40 nations pinpointed *Enterococcus* as a major pathogen associated with hospital-acquired diseases [226]. *E. faecium* was identified as the second and third most common pathogen associated with bloodstream and urinary catheters in patients from Germany [224]. Clinical surveillance at a major women’s specialty hospital in southwestern China identified *E. faecalis* as the secondary etiological agent in postoperative infections following gynecological surgeries, ranking only behind *S. aureus* in incidence frequency [227].

#### 4.1.1. *Enterococcus*-Associated Urinary Tract Infections

Urinary tract infections represent a persistent global health challenge that affects populations of all ages and sexes. Although *E. coli* remains the predominant uropathogen, responsible for approximately four-fifths of all cases, *Enterococcus* spp. have gained recognition as important secondary etiological agents, collectively accounting for a substantial proportion of the remaining infections [228].

Recent epidemiological investigations have documented an increasing role of *Enterococcus* spp. in the pathogenesis of urinary tract infections. A 2023 meta-analysis by Noor et al. established multiple comorbidities and iatrogenic factors predisposing patients to enterococcal UTIs, notably diabetes mellitus, chronic renal impairment, malignancy, cerebrovascular accidents, and instrumentation with urinary catheters. The study further revealed that extended hospitalization (>14 days) and prior broad-spectrum antimicrobial therapy were significantly correlated with vancomycin-resistant enterococcal urinary infections [229]. Another study on a 72-year-old patient with urinary tract infection symptoms caused by *Enterococcus avium* and *Escherichia coli* showed incomplete resolution with initial antibiotic therapy (cefuroxime), as persistent haematuria was later attributed to a bleeding bladder lesion and staghorn kidney stones. Full recovery was achieved only after surgical resection of the lesion and removal of the stones, thus emphasizing that management of infections caused by rare enterococci may require both targeted antimicrobial therapy and procedural intervention [230].

The uropathogenicity of *Enterococcus* spp. stem largely from two key biological attributes: their capacity for biofilm development and for secreting virulence determinants. Biofilm architecture enables these microorganisms to evade host immune clearance while simultaneously enhancing antimicrobial tolerance [223]. This complex developmental process progresses through distinct phases: beginning with surface adherence and progressing to clustered cellular aggregation, followed by extracellular polymeric substance synthesis to form a mature biofilm structure, and culminating in cellular detachment for systemic spread. Urinary catheter surfaces serve as optimal substrates for *Enterococcus* biofilm development, often leading to catheter-associated urinary tract infections [231]. The pathogenic mechanisms of these biofilm-forming uropathogenic strains operate through several synergistic pathways: (1) generation of dense extracellular polymeric matrices that impede antimicrobial penetration, (2) horizontal acquisition of genetic resistance determinants, and (3) polymicrobial colonization with species such as *E. coli* that can potentiate virulence through immunomodulatory effects and synergistic pathogenicity enhancement [232].

#### 4.1.2. *Enterococcus*-Associated Bloodstream Infections

Bloodstream infections caused by *Enterococcus* spp. (EBSIs) have become a critical healthcare challenge, presenting considerable clinical and therapeutic difficulties. While these bacteria normally colonize the intestinal tract and contribute to gut microbial balance, they can translocate across the intestinal epithelium under specific conditions, resulting in systemic infections with severe clinical consequences [233]. 

The pathogenesis of *Enterococcus*-associated bloodstream infections (EBSIs) is strongly linked to immunosuppression resulting from comorbidities, prolonged hospitalization, or invasive medical interventions. Key predisposing factors include: immunocompromised states secondary to chronic diseases (e.g., diabetes mellitus, inflammatory bowel disorders), iatrogenic factors such as indwelling catheters or prolonged intravenous access, and extended inpatient care duration, which increases exposure to nosocomial pathogens [54]. 

Clinical data identify *E. faecalis* and *E. faecium* as the principal etiological agents of enterococcal bloodstream infections. Although *E. faecalis* has a higher incidence, *E. faecium* infections typically present greater clinical severity. This heightened virulence profile stems largely from *E. faecium*’s enhanced capacity to acquire antimicrobial resistance, which frequently complicates therapeutic management and may lead to poorer patient outcomes [234]. Untreated enterococcal bloodstream infections can progress to severe systemic complications, including inflammatory endocardial disease, persistent bacteremia, central nervous system infections, bone marrow inflammation, and systemic inflammatory response syndrome. Research indicates that intestinal microbial dysbiosis and subsequent enterococcal overgrowth facilitate bacterial translocation across mucosal barriers, enabling hematogenous dissemination to distant anatomical sites and the development of these high-mortality conditions [235,236,237]. Recent genomic investigations by Chaguza et al. have elucidated the contribution of hereditary determinants to the virulence potential of *E. faecalis*. Their analysis revealed that the bacterial genotype accounts for 30–40% of the observed variability in clinical manifestations, significantly influencing both tissue tropism (particularly extraintestinal colonization) and epidemiological distribution patterns (with distinct strain predilection for nosocomial transmission). These findings underscore the fundamental role of microbial genomics in determining pathogenic behavior [238]. 

In addition to the predominant *E. faecalis* and *E. faecium*, several less common *Enterococcus* spp. have been implicated in bloodstream infections, including *E. casseliflavus*, *E. durans*, *E. hirae*, *E. avium*, and *E. raffinosus*, with most cases representing rare clinical occurrences [239,240]. Notably, *E. casseliflavus* is intrinsically resistant to vancomycin, necessitating alternative therapeutic approaches. For example, A clinical review of two *E. casseliflavus* bacteremia cases in hemodialysis patients with chronic renal failure highlighted the role of underlying comorbidities in facilitating invasive infection. Both cases did not respond to vancomycin therapy but responded rapidly to targeted daptomycin-based combination therapy (with imipenem or cefepime), underscoring the therapeutic potential of combination regimens and the need for heightened awareness of intrinsically vancomycin-resistant *Enterococcus* in bloodstream infections complicating chronic illness [241]. Another reported case of concomitant *Enterococcus casseliflavus* bacteremia and *Raoultella planticola* biliary colonization occurred in a 74-year-old male with pancreatic adenocarcinoma and ascending cholangitis following biliary stent placement. Blood cultures confirmed *E. casseliflavus* (intrinsically vancomycin-resistant via VanC), while bile cultures yielded *E. casseliflavus*, *R. planticola*, *Enterobacter cloacae*, and *Candida albicans*. The patient improved with targeted therapy including cefepime, daptomycin, metronidazole, and fluconazole, underscoring the opportunistic pathogenic potential of these rare organisms in gastrointestinal malignancy and the importance of accurate species identification to guide therapy [242]. Current evidence supports the efficacy of β-lactams (particularly ampicillin), lipopeptides (daptomycin), and carbapenem-class antibiotics against this species [243].

#### 4.1.3. *Enterococcus*-Associated Central Nervous System (CNS) Infections

Enterococcal CNS infections predominantly affect vulnerable patient populations with neurological compromise, including those with recent neurosurgical interventions, structural CNS abnormalities (e.g., ventriculoperitoneal shunts, traumatic defects), and pre-existing neurological disorders. While enterococcal meningitis and ventriculitis (EMV) remain uncommon, their incidence has risen notably in pediatric inpatient settings, where invasive devices and compromised barriers facilitate microbial CNS invasion [244].

A decade-long retrospective analysis by Schnapp et al. systematically evaluated pediatric EMV cases, characterizing etiological patterns, predisposing conditions, and therapeutic challenges. The investigation revealed two predominant risk categories: iatrogenic factors, particularly cerebrospinal fluid diversion hardware (ventricular shunts and drainage systems), and developmental vulnerabilities, including premature birth. Notably, antimicrobial resistance has emerged as a critical concern, with vancomycin nonsusceptibility observed in one-fifth of the clinical strains. Rare but clinically important reports expand the pathogenic spectrum beyond *E. faecalis* and *E. faecium*. For example, the first documented case of *E. casseliflavus* meningitis occurred in an immunosuppressed elderly patient, where intrinsic vancomycin resistance precluded standard therapy; however, meropenem plus ampicillin–sulbactam achieved recovery [245]. Likewise, *E. gallinarum*, a typically poultry-associated species, caused meningitis in a 53-year-old man with alcohol abuse, who was successfully treated with high-dose ampicillin and gentamicin [246]. In pediatrics, *E. gallinarum* group meningitis was reported for the first time following bowel perforation from a ventriculoperitoneal shunt; its intrinsic vancomycin resistance necessitated a 6-week targeted intravenous regimen, highlighting the need for culture-guided therapy in shunt-related CNS infections [247]. A 2016 clinical investigation by Patel and colleagues demonstrated superior therapeutic outcomes with direct intraventricular vancomycin injection for enterococcal meningitis compared with conventional intravenous administration. This finding underscores the critical influence of the administration route on the treatment efficacy for central nervous system infections [248].

#### 4.1.4. *Enterococcus*-Associated Animal and Zoonotic Infections

Some enterococci, especially *E. faecalis* and *E. faecium*, continue to be recognized as important pathogens in both animal health and zoonotic transmission [225,231]. In animals, these enterococcal species are associated with conditions such as UTI, mastitis, wound infections, and septicemia [225]. Companion animals, including dogs and cats, frequently harbor multidrug-resistant (MDR) strains, with studies reporting high rates of resistance to antimicrobials such as tetracycline and erythromycin [249]. The close interaction between pets and humans increases the risk of bidirectional transmission, making companion animals important reservoirs of resistant enterococcal strains [54]. Livestock farming further amplifies these concerns, as *E. faecalis* and *E. faecium* are prevalent in poultry and swine, often demonstrating less sensitivity to critical antibiotics such as vancomycin [225]. For example, *E. cecorum*, an emerging pathogen in poultry, is associated with systemic infections and skeletal disorders, resulting in substantial economic losses [250]. In pig farms, resistant strains not only compromise animal health but also contribute to the dissemination of AMR in farming environments [251]. Extensive use of antimicrobials in agriculture exerts selective pressure, enabling the survival and spread of resistant strains. These resistant strains can contaminate foods of animal origin, thereby increasing the risk to humans through dietary exposure [6,44]. The impact of enterococcal infections is not limited to terrestrial animals. Wildlife, particularly marine species such as sea turtles, has also been affected, with cases of septicemia and osteomyelitis caused by *E. faecalis* linked to environmental contamination [252]. This highlights the adaptability of enterococci, which can persist across diverse ecosystems and pose risks to multiple species.

Zoonotic infections caused by pathogenic enterococci pose significant challenges, particularly for immunocompromised individuals, where conditions such as bacteremia and endocarditis may become life-threatening [253]. For instance, a rare case of *E. casseliflavus* endophthalmitis following a pig-related eye injury illustrates its zoonotic potential; the organism’s intrinsic vancomycin resistance limits first-line empiric therapy, but intravenous ampicillin and dexamethasone improved symptoms, and systemic linezolid enhanced visual acuity, although intravitreal amikacin remains an important option when oral linezolid is insufficient [254]. Similarly, a recent report described a diabetic foot ulcer infection caused by *E. avium* in a patient with documented poultry exposure, complicated by osteomyelitis and recurrent bacterial colonization. This case required combined surgical intervention and tailored antibiotic therapy guided by microbial monitoring, underscoring the need to consider zoonotic *Enterococcus* species in clinical diagnoses, especially among immunocompromised or chronically ill patients [255]. The increasing prevalence of such infections highlights the urgent need for a One Health approach, fostering collaboration across human health, veterinary medicine, and environmental management to address the complex interactions that facilitate zoonotic transmission of enterococci [225].

#### 4.1.5. Foodborne *Enterococcus* Infections

Enterococci serve as important microbial indicators in food safety systems, reflecting possible fecal contamination and inadequate sanitary controls during manufacturing [23]. Although they are not typical causative agents of foodborne disease, their presence in food products suggests potential co-contamination with enteric pathogens, including *Salmonella*, *E. coli*, and *L. monocytogenes*, which can trigger acute foodborne disease outbreaks [256]. A 2024 Kenyan surveillance study found *Enterococcus* contamination in 50% of sampled infant foods, indicating widespread fecal pollution in domestic food-preparation environments [257]. While epidemiological analysis failed to identify a singular contributing factor for this contamination level, the frequent detection of these indicator organisms strongly suggests inadequate hygiene protocols during food production, storage, or handling. Although most enterococci rarely cause foodborne illness, their continued presence requires the immediate adoption of improved food safety measures to prevent possible pathogen transmission [258].

### 4.2. Virulence Factors Associated with Enterococcus spp.

*Enterococcus* spp. employ diverse virulence determinants that increase their pathogenicity in their hosts. While some virulence determinants are strain-specific, others are conserved across strains within the genus. These determinants often facilitate host tissue invasion, immune evasion, and intracellular persistence, enabling colonization of multiple anatomical sites in the infected host. Key virulence mechanisms include: host cell adhesion (mediated by *Asa1, efaA*, and *ace*), biofilm formation (*esp*, *gelE*), cytolytic activity (*cylA*), and tissue penetration (*hylA*). Collectively, these factors subvert host defenses, damage cellular structures, and promote enterococci infectivity [227].

Asa1-encoded aggregation substance (AS) is a multifunctional virulence factor that mediates biofilm development and conjugative plasmid transfer, thereby enhancing the dissemination of antimicrobial resistance [101]. It also evades the immune system by binding to neutrophils independently of opsonins, impairs phagocytic clearance, and enhances macrophage adhesion, thereby promoting intracellular persistence [259]. This role in both horizontal gene transfer and host defense subversion underscores *AS* as a critical pathogenicity determinant in *E. faecalis* infections.

Enterococcal surface protein (*Esp*) is a critical virulence determinant that facilitates biofilm formation, host tissue adhesion, and the transfer of antibiotic resistance determinants [95]. Experimental studies in murine models have demonstrated the dual pathogenicity of *Esp*, which enables intestinal colonization and cardiac valve adherence, promotes endocarditis pathogenesis, and is indispensable for biofilm formation. While *Esp*-positive *E. faecalis* strains consistently form biofilms within their niches, *Esp*-negative variants are unable to form biofilms or establish structured communities. These findings position *Esp* as a master regulator of enterococcal persistence and dissemination [228,260]. Similarly, the *efaA* virulence gene, which exists as strain-specific variants *efaAfs* (*E. faecalis*) and *efaAfm* (*E. faecium*), is highly linked with endocarditis pathogenesis [21,261]. The *EfaA* surface antigen functions as a critical adhesin in *E. faecalis*, exhibiting host-specific expression patterns. This manganese acquisition protein demonstrates serum-inducible characteristics, showing preferential production during blood culture compared with standard media. As a component of the manganese transport system, *EfaA* facilitates essential nutrient uptake during bloodstream infection when manganese demand is elevated. Its expression is tightly regulated by the *EfaR* repressor protein, which downregulates *EfaA* production under manganese-replete conditions [262]. Dysfunctional *EfaR* regulation not only disrupts manganese homeostasis but also impairs biofilm formation and intracellular survival within phagocytic cells. This has been documented in *Enterococcus*-associated endocarditis, where *E. faecalis* adheres to the cardiac endothelium and forms antibiotic-resistant biofilms due to *EfaA*, which is essential for both biofilm maturation and persistent colonization of heart tissue [263]. These adaptive mechanisms significantly increase bacterial virulence, directly contributing to *E. faecalis* capacity to establish and maintain endocardial infections [223].

The *Ace*-encoded adhesin mediates *E. faecalis* attachment to host collagenous matrices, facilitating the colonization of biomedical implants and host tissues [259]. While both *Ace* and *Acm* proteins exhibit structural homology with microbial surface components that recognize adhesive matrix molecules (MSCRAMMs), *Ace* proteins demonstrate unique thermosensitive binding properties, with optimal collagen affinity at 46 °C, a temperature exceeding physiological levels [22,264]. This paradoxical behavior may reflect competitive interactions with gelatinase, a collagen-degrading enzyme produced by certain *E. faecalis* strains that counteract adhesion [265]. Another important virulence determinant often found in clinical *E. faecalis* (specifically *E. faecalis* subsp. *liquefaciens* is the *GelE* metalloprotease. *GelE* subverts innate immunity by targeting the cleavage of the complement component C3, which impairs opsonization and subsequent phagocytic clearance by neutrophils and macrophages, enabling immune evasion [266].

The *cylA* gene is part of a six-gene operon (*cylLL*, *cylLS*, *cylM*, *cylB*, *cylA*, and *cylI*) that coordinately regulates the biosynthesis and activation of cytolysin, a potent exotoxin capable of lysing both prokaryotic and eukaryotic cells [267]. This toxin contributes to *Enterococcus* pathogenicity through multiple mechanisms, including host cell lysis, which targets erythrocytes (causing anemia), destroys leukocytes and macrophages (impairing immune defenses), and induces parenchymal tissue damage in vital organs [95,268]. They are also prevalent in clinical strains, and are correlated with severe infections, promoting tissue necrosis at infection sites and amplifying inflammatory cascades through the release of DAMPs [266].

### 4.3. Antibiotic Resistance in Enterococcus spp.

The antimicrobial resistance (AMR) trajectory of *Enterococcus* spp. predate their formal taxonomic classification [269]. Initial therapeutic strategies relied on β-lactam monotherapy, which progressively lost efficacy due to the development of resistance. This prompted the adoption of synergistic β-lactam/aminoglycoside regimens, although their use was similarly compromised by emerging resistance mechanisms. The Subsequent dependence on vancomycin as a last-line agent was undermined by the first documented vancomycin-resistant *Enterococcus* (VRE) isolates in 1989 [8]. Alarmingly, each newly introduced anti-enterococcal agent has experienced rapid emergence of resistance, creating an escalating therapeutic crisis in the management of these infections [44,113].

*Enterococcus* spp. exhibit two distinct antimicrobial resistance (AMR) profiles: intrinsic (encoded by conserved chromosomal genes present across all strains) and acquired (mediated by mobile genetic elements or mutations). The innate resistance spectrum of these bacteria includes β-lactams (cephalosporins, penicillins), aminoglycosides, and macrolides [269,270]. For example, *E. gallinarum* and *E. casseliflavus* are intrinsically resistant to vancomycin and have shown high-level aminoglycoside resistance [271]. These vancomycin-resistant enterococci (VRE) have been isolated from animal feces and foods [272,273]. Acquired resistance, however, arises primarily through horizontal gene transfer via plasmids or transposons, or via mutations with high transfer potential—a process predominantly facilitated by conjugation [274]. A study on meats from Ghana detected *E. hirae*, *E. casseliflavus*, *E. gallinarum*, *E. thailandicus*, and *E. lactis* carrying multiple resistance genes, including those for tetracyclines (*tet(M)*, *tet(L)*, *tet(S)*), macrolides (*erm(B)*, *erm(T)*), and aminoglycosides (*aac(6’)-Ii*, *aph(3’)-III*), among others. Mobile genetic elements, such as plasmids (rep1, rep2, repUS43) and transposons (Tn917, Tn6009), were found to co-occur with these genes, suggesting a potential for horizontal gene transfer [60].

The common genes that confer AMR in *Enterococcus* include the following:

***blaZ* gene:** The blaZ gene, which mediates β-lactam resistance through enzymatic antibiotic inactivation, has been increasingly identified in *Enterococcus* spp. despite its historical association with *S. aureus* [98,231]. This horizontal gene transfer event suggests the intergeneric dissemination of resistance determinants. Epidemiological surveillance has detected *blaZ*-carrying *Enterococcus* strains across diverse reservoirs, including human clinical isolates, livestock-associated samples, and food production chains [275]. *blaZ* expression is often regulated by a coordinated system involving *blaI* (repressor), *blaR1* (signal transducer), and *blaR2* (auxiliary regulator) proteins, which collectively modulate β-lactamase production in response to antibiotic exposure [276,277]. The *blaZ* gene was identified in 9.6% of *Enterococcus* isolates from clinical samples [278] and in 48.1% of strains from fish and seafood [279]. Similarly, strains from nestling ospreys (*Pandion haliaetus*) and other wildlife species harbor *blaZ* resistance genes, highlighting the widespread prevalence of this resistance gene across diverse environments and its role in the dissemination of antibiotic resistance [280].

***mecA* gene:** The *mecA* gene encodes penicillin-binding protein 2a (PBP2a), a modified transpeptidase with reduced binding affinity for β-lactam antibiotics, thereby conferring resistance to this drug class [269]. Although it is historically associated with *Staphylococcus* spp, the increasing detection of *mecA* in diverse *Enterococcus* strains provides compelling evidence of interspecies horizontal gene transfer, a phenomenon that significantly expands the antibiotic resistance potential of these opportunistic pathogens [269,281]. Emerging evidence indicates that *mecA*-positive *Enterococcus* strains may drive nosocomial infections through parallel pathogenic mechanisms to methicillin-resistant *Staphylococcus* spp. A recent clinical survey revealed methicillin resistance in 85% of the analyzed enterococcal isolates, with 95.5% testing positive for *mecA* carriage. Furthermore, while *mecA* dynamics in enterococci remain understudied, these findings suggest its potential role in propagating methicillin resistance across bacterial populations via horizontal gene transfer—a concerning prospect for antimicrobial resistance containment in healthcare environments [33].

***Van* genes:** These genetic determinants mediate glycopeptide resistance through enzymatic alteration of peptidoglycan precursors, specifically by modifying the D-alanyl-D-alanine target site to reduce vancomycin binding affinity. The resulting structural changes in cell wall biosynthesis render glycopeptide antibiotics, such as vancomycin, therapeutically ineffective [277]. Among the various van operons, *vanA* and *vanB* are the most prevalent, with *E. faecalis* and *E. faecium* being their primary carriers [21]. *VanA* and *vanB* confer resistance by modifying the bacterial peptidoglycan, replacing the D-Ala-D-Ala terminal with D-Ala-D-Lac, which prevents vancomycin from binding and inhibiting cell wall synthesis. The *VanC*-mediated resistance mechanism operates through a distinct biochemical pathway, substituting the conventional D-Ala-D-Ala peptidoglycan precursor with D-Ala-D-Ser at an approximate 3:1 ratio. This genetic determinant is primarily observed in motile *Enterococcus* spp., including *E. casseliflavus*, *E. gallinarum*, and *E. flavescens*, representing an intrinsic resistance phenotype that differs fundamentally from acquired vancomycin resistance mechanisms [37]. While vancomycin resistance determinants, such as *vanD*, *vanE*, *vanG*, *vanL*, *vanM*, and *vanN*, occur sporadically in *Enterococcus*, surveillance of 40 commercial probiotic formulations for livestock revealed interesting findings. The *vanA* gene, a high-level vancomycin resistance marker, was detected in these products, with its plasmid- and transposon-borne location facilitating potential horizontal transfer to pathogenic species. This mobility underscores the risk of resistance dissemination through the use of probiotics in agriculture [282].

Studies have reported that most nasal *Enterococcus* isolates harbor multidrug resistance, including high-level aminoglycoside and glycopeptide resistance. Vancomycin-resistant enterococci (VRE), particularly *E. faecium* with *VanA* and *VanB* genotypes, are prevalent in healthcare settings and are increasingly detected in nasal samples of patients and in animal reservoirs (e.g., parrots and rabbits) [32]. Altered nasal microbiota composition, involving *Enterococcus* species, is associated with chronic rhinosinusitis and nasal polyps, where impaired mucosal barrier function and reduced antimicrobial peptides promote bacterial invasion and inflammation. Nasal colonization with multidrug-resistant (MDR) *Enterococcus*, especially VRE, is linked to increased risk of invasive infections in immunocompromised and ICU patients [34]. Surveillance data from England show a plateau in *Enterococcus* bloodstream infections but rising vancomycin resistance in *E. faecium* isolates, emphasizing ongoing clinical challenges. Furthermore, co-colonization of the nasal cavity with *S. aureus* facilitates the exchange of resistance genes, thereby potentiating nosocomial outbreaks [283]. 

***Tet* genes:** Tetracycline resistance in *Enterococcus* is mediated by diverse genetic determinants encoding two primary resistance mechanisms: (1) ribosomal protection proteins (e.g., *Tet(M*), *Tet(O*), and *Tet(S)*) that safeguard the translation machinery, and (2) efflux pumps (e.g., *Tet(K)*, and *Tet(L)*) that actively export tetracyclines from bacterial cells [269,284]. Ribosomal protection mediated by *tet(M)* represents the predominant tetracycline resistance mechanism in *Enterococcus*, although *tet(L)*-encoded efflux also contributes substantially to resistance profiles. Notably, the co-occurrence of *tet(M)* and *tet(L)* confers synergistic high-level resistance [285]. Both genes exhibit mobility and can be transferred interbacterially via plasmid-independent mechanisms, thereby facilitating rapid dissemination of resistance even in the absence of conjugative elements. The dissemination of tetracycline resistance genes in *Enterococcus* is facilitated primarily by conjugative transposons of the Tn916/Tn1545/Tn5397 family [269]. Among these mobile genetic elements, Tn916 and Tn5397 have the highest epidemiological prevalence, whereas Tn1545 occurs less frequently and is typically found only in association with other transposons of this family [285,286].

***Erm* genes:** Macrolide resistance in *Enterococcus* is mediated by rRNA methylase enzymes encoded by *erm* genes (particularly *erm(A)*, *erm(B)*, and *erm(C)*), which dimethylate the 23S rRNA target site. This modification sterically hinders macrolide binding, conferring resistance to antibiotics such as erythromycin. Notably, these resistance determinants exhibit high mobility through transposon-mediated horizontal gene transfer. *Enterococcus* spp demonstrate additional macrolide resistance through *mef*- and *msr*-encoded efflux pumps [287]. *E. faecalis* exhibits intrinsic resistance to lincosamides (clindamycin) and streptogramins (quinupristin-dalfopristin) via the *lsa* efflux determinant. This species-specific resistance mechanism is chromosomally encoded in *E. faecalis* but absent in other enterococci, highlighting unique evolutionary adaptations within this taxon [37].

**Aminoglycoside-modifying enzymes-encoding genes:** Aminoglycoside resistance in *Enterococcus* is mediated by enzymatic modification through three primary gene families: aminoglycoside phosphotransferases (*aph*), aminoglycoside acetyltransferases (*aac*), and aminoglycoside nucleotidyltransferases (*ant*). These enzymes structurally alter antibiotics (e.g., gentamicin, kanamycin, and streptomycin) via phosphorylation, acetylation, or nucleotidylation, respectively. Such modifications sterically hinder drug-target interactions at the 30S ribosomal subunit, thereby conferring high-level resistance [44,288]. Enterococci possess a diverse array of *aph* and *ant* resistance determinants that specifically target different classes of aminoglycosides. The *aph*(3’)-III variant confers resistance to kanamycin and neomycin through phosphorylation, whereas *ant*(6)-Ia mediates streptomycin and tobramycin resistance via nucleotidylation. These genetic elements frequently reside on mobile platforms, particularly conjugative plasmids and transposons, enabling efficient horizontal dissemination among bacterial populations through mechanisms such as conjugation and transposition [135,289]. The selected *Enterococcus* spp., including *E. faecium*, *E. durans*, and *E. hirae*, encode aminoglycoside acetyltransferases (*AACs*) that confer resistance to specific aminoglycoside subclasses. Additionally, *E. faecium* harbors *EfmM* methyltransferase, a unique resistance determinant that confers high-level resistance to kanamycin and tobramycin by modifying the ribosomal target [290,291]. 

All *Enterococcus* spp. exhibit intrinsic low-level aminoglycoside resistance due to limited drug uptake across their cell wall. However, synergistic bactericidal activity occurs when aminoglycosides are combined with cell wall-targeting agents (e.g., vancomycin or ampicillin). These combinations enhance aminoglycoside penetration, overcoming the innate resistance barrier and effectively killing enterococcal cells [44,292].

***Enterococcus* biogenic amines (BA)**: Biogenic amines are nitrogenous compounds generated through the enzymatic decarboxylation of amino acids, which are naturally present in fermented foods (cheese, sausages, beer, wine). *Enterococcus* spp., which are prevalent in food matrices, contribute to BA accumulation and are associated with foodborne intoxication [293]. While tyramine is the most studied BA in enterococci, recent evidence confirms its capacity to synthesize additional amines (histamine, putrescine, tryptamine, phenylethylamine) via histidine/tyrosine decarboxylase activity during dairy fermentation [294]. These molecules, synthesized by BA-producing *Enterococcus* strains, function as neuroactive agents (e.g., norepinephrine modulation) and as virulence enhancers, exhibiting greater pathogenicity than non-producers [114,295]. A study examining quorum-sensing-mediated biogenic amine production in *Sanchuan* ham identified *E. faecium* as the primary tyramine producer. Key findings revealed that elevated temperatures and reduced sodium chloride concentrations significantly increase tyramine biosynthesis in this strain. Tyrosine serves as the exclusive precursor for tyramine generation, whereas the dipeptide alanyl-leucine functions as a quorum-sensing signal that upregulates tyrosine decarboxylase expression and modulates tyramine output [296]. 

***Enterococcus* sex pheromones**: Microbial-derived peptide pheromones play pivotal roles in facilitating interbacterial communication and genetic exchange. In *Enterococcus*, these signaling molecules function as chemoattractants, inducing donor–recipient cell proximity to facilitate conjugative plasmid transfer [297,298]. Chromosomally encoded sex pheromone genes enable enterococcal strains to acquire exogenous genetic material through this highly efficient conjugation mechanism. Recent genomic investigations have elucidated the role of plasmid-encoded peptide mediators in facilitating horizontal gene transfer between *Enterococcus* strains. Tomita et al. characterized type A pheromone plasmids in vancomycin-resistant enterococci (VREs) isolated from both human and animal reservoirs. These mobile genetic elements respond to cAD1 pheromone signaling, initiating conjugative transfer of the *vanA* resistance determinant to recipient cells [265]. Emerging research has identified pCF10-like pheromone-responsive plasmids as key vectors in the interspecies dissemination of oxazolidinone resistance determinants between *Enterococcus* and *Staphylococcus* spp. [299,300]. This conjugative system exhibits remarkable specificity, wherein donor-derived plasmids exclusively recognize and respond to complementary pheromones secreted by recipient cells, thereby activating the expression of plasmid-encoded transfer machinery [301]. Pheromone-receptor binding in donor cells triggers AS gene upregulation, initiating plasmid transfer via aggregation substance-mediated conjugation [302,303]. In addition to genetic exchange, these signaling molecules exhibit pleiotropic effects, including immune modulation via chemotaxis of phagocytic cells, proinflammatory activity via cytokine cascade activation, and oxidative stress via superoxide production, which can cause cellular toxicity [75,266].

## 5. Beneficial Enterococci: Potential in the Health and Agri-Food System Versus Legislation

The regulatory framework governing the application of *Enterococcus* probiotics in food and healthcare has significant jurisdictional variation. National authorities typically oversee the use of probiotics through dedicated food and drug regulatory bodies. For example, in the United States, dual oversight is carried out by the Food and Drug Administration (FDA) for safety/efficacy and the Department of Agriculture (USDA) for agricultural applications. In the European Union, EFSA (European Food Safety Authority) approval is required for health claims, while in Canada, Canada’s Natural Health Products Directorate regulates probiotic supplements [304].

The European Union employs a dual regulatory framework for probiotics in food and feed applications, overseen by the European Food Safety Authority (EFSA) and the European Commission. EFSA’s mandate includes conducting scientific risk assessments, evaluating the safety and efficacy of probiotic strains for human consumption and animal feed, and implementing a streamlined safety assessment system for microorganisms used in food/feed production. Introduced in 2005, this system allows well-characterized microbial strains with established safety profiles to undergo simplified authorization processes, whereas novel strains require comprehensive evaluation [305]. The EU enforces stringent microbiological criteria for food safety through regulations such as Regulation (EC) No 2073/2005, which outlines microbiological standards for foodstuffs. While *Enterococcus* spp. are not directly classified as pathogens of concern, their presence is monitored as a hygiene indicator or as a marker of faecal contamination in various food products, including cheese, meat, and water. This regulation ensures that food producers maintain acceptable microbial safety levels, with *Enterococcus* spp. playing a key role in identifying potential contamination risks [116].

In addition to food safety, the use of *Enterococcus* spp. as probiotics is governed by EU guidelines to ensure public health protection. The EFSA provides comprehensive guidance on the safety assessment of bacterial strains used as food/feed additives or as probiotics. Strains intended for such purposes must undergo a QPS assessment, in which critical safety factors, including AMR, toxin production, and virulence factors, are carefully evaluated [306]. This approach ensures that only nonpathogenic, safe strains are used in food products and animal feed. Moreover, Regulation (EC) No 1831/2003 specifically regulates feed additives, including probiotic *Enterococcus* strains. According to this regulation, only those strains listed in the EU Register of Feed Additives that have been proven safe for animals, humans, and the environment are authorized for use. Additionally, these strains must not contribute to the dissemination of antibiotic resistance genes, aligning with broader EU efforts to curb the spread of AMR [307].

In fermented foods such as cheese, *Enterococcus* spp. are often considered part of the natural microbiota and are permitted within certain limits. However, it remains essential for food producers to certify that the strains used in fermentation are nonpathogenic and do not harbor transferable AMR genes. This is part of a greater effort to maintain food safety standards and reduce the risk of AMR transmission across the food chain [305]. *Enterococcus* spp., particularly *E. faecalis* and *E. faecium*, are also utilized as indicators of fecal contamination in water testing. European countries adhere to the EU Water Framework Directive (Directive 2000/60/EC) and Directive 98/83/EC, which regulate the quality of water intended for human consumption. These directives specify maximum allowable levels of *Enterococcus* spp. for various water types, including drinking and bathing water, to protect public health [116].

The European Antimicrobial Resistance Surveillance Network (EARS-Net), alongside EFSA, monitors trends in AMR in *Enterococcus* isolates from humans, animals, and food products. *E. faecalis* and *E. faecium* are of particular concern because of their resistance to critical drugs such as vancomycin. To address this issue, the EU has implemented measures to reduce AMR, such as restricting the use of antimicrobials in agriculture and enforcing rigorous hygiene standards in food production [306]. While these EU regulations establish a unified framework, individual countries often adopt additional, more specific guidelines or monitoring programs to address local needs and concerns. For example, Denmark and the Netherlands have emerged as leaders in AMR surveillance, implementing stringent measures to control the spread of resistant *Enterococcus* strains across health care settings, agriculture, and food production [116]. These country-specific initiatives complement EU-wide regulations and further mitigate the risks posed by AMR. 

Regulatory agencies across Africa oversee the use of probiotics in food, feed, and health products to ensure safety and compliance. In Nigeria, the National Agency for Food and Drug Administration and Control (NAFDAC) regulates probiotics under the NAFDAC Act, Cap N1, LFN 2004, and implements measures to combat AMR, including banning antibiotics as growth promoters in livestock. To support sustainable production, NAFDAC promotes alternatives such as probiotics, prebiotics, enzymes, and organic acids. Additionally, strict regulations on the use of *Enterococcus* in animal feed align with European standards, ensuring safety and mitigating health risks [308].

The Qualified Presumption of Safety (QPS) system streamlines regulatory approval for microbial strains by exempting well-characterized groups from exhaustive safety evaluations, provided that no disqualifying traits are identified. For microorganisms lacking QPS status, such as certain *Enterococcus* spp., mandatory full safety assessments are required, including taxonomic verification, pathogenic potential analysis, intended use context, and a review of historical safety data [307,309].

## 6. Bioengineering of *Enterococcus* spp.

The bioengineering of *Enterococcus* spp., particularly *E. faecalis* and *E. faecium*, has attracted increasing interest because of their probiotic potential and industrial applications. These microorganisms, known for their resilience and ability to thrive in harsh environments, can be genetically modified to improve performance, benefitting healthcare, food, and agriculture. Some of the modern and emerging techniques in bioengineering for improving *Enterococcus* strains include the following:

**CRISPR Genome Editing:** Clustered Regularly Interspaced Short Palindromic Repeats (CRISPR) is a powerful tool for genome editing, allowing precise modifications to DNA in living organisms. This revolutionary technique allows for precise, targeted alterations in the *Enterococcus* genome. CRISPR can be used to knock out specific genes, insert new genes, or modify existing genes to enhance probiotic characteristics such as antimicrobial production, stress tolerance, or adhesion to intestinal cells [310].

This precision makes CRISPR an ideal tool for creating strains with tailored properties for applications in medicine and the food industry. For example, a study involving the multidrug-resistant *E. faecium* strain E745 utilized CRISPR-Cas9 to generate a deletion mutant in the *lacL* gene, which encodes the large subunit of *E. faecium* β-galactosidase [311]. Another study created *E. faecium* mutants in which the CRISPR-Cas12a system lacked the *acpH*, *treA*, and *lacL* genes. These genes are involved in phosphopantetheinylation, are responsible for trehalose-6-phosphate hydrolase activity, and encode the large subunit of β-galactosidase [312]. This method also reduced the time required for genome modification to 3 weeks, highlighting the efficiency of CRISPR. 

**Enterocin Engineering:** Enterocins are antimicrobial peptides produced by *Enterococcus* and other LAB. Through bioengineering, bacterial strains can produce novel or enhanced bacteriocins [313]. For example, *Pichia pastoris* was genetically modified to produce a recombinant mutant capable of producing enterocin P at high concentrations [195]. In another study, *E. coli* strains were engineered to secrete two specific enterocins, enterocin A (EntA) and enterocin B (EntB), and the engineered *E. coli* strains exhibited strong antimicrobial activity against the targeted *Enterococcus* spp. [314].

**Phage Therapy and Engineering:** With increasing interest in antimicrobial resistance, engineered bacteriophages are being explored as tools to target and eliminate pathogenic *Enterococcus* strains [315]. Through genetic modifications, phages can be made more efficient or specific, by helping to control infections while preserving beneficial strains.

## 7. Limitations

Studies have continuously demonstrated the beneficial and multifaceted effects of some strains of enterococci in health agrifood systems. However, due to multiple safety concerns, the application of some beneficial enterococci strains as feed additives or probiotics has not been explicitly studied, despite their antimicrobial, health, and nutritional potential. Among others, major limitations and uncertainties include the potential dissemination of cytotoxic, multidrug-resistant, and virulence determinants through horizontal gene transfer and evolution. Also, the diversity of regulatory frameworks across countries and regions makes pre-market safety evaluations highly heterogeneous, discriminatory, and inconsistent. While we identified limitations in extrapolating from preliminary in vitro and animal data to clinical or human use of beneficial enterococci, most studies did not explicitly differentiate food-derived, commensal, environmental, and clinical enterococcal strains used in their studies. 

## 8. Conclusions

Multiple strains of beneficial enterococci are increasingly used as protective cultures, alternatives to antibiotics, and probiotics to control pathogens, mitigate disease, modulate the microbiome, and support overall host health. They also support food fermentation and safety, enhance sensory properties, and produce bioactive compounds such as bacteriocins with strong pathogen-inhibitory activity and multifarious health benefits. Despite their advantages in health and agrifood systems, their association with healthcare-associated infections and the spread of antimicrobial resistance raises concerns about their safety. These risks underscore the need for stringent safety evaluations before their use as probiotics, alternatives to antibiotics, or protective cultures.

The genus *Enterococcus* demonstrates a paradoxical duality in microbial ecology, functioning both as opportunistic pathogens in healthcare settings and as beneficial probiotics in agrifood systems and health. This dichotomy is exacerbated by their frequent carriage and dissemination of AMR genes and virulence factors, posing significant public health challenges and making them both valuable and potentially hazardous across food, clinical, and environmental landscapes. The complexity of their safety profile underscores the need for thorough risk assessments and cautious, strain-by-strain rigorous evaluation and approval prior to their application in the agrifood and health sectors. Regulatory frameworks across countries and regions of the world should be consistent and nondiscriminatory and must evolve alongside advances in biotechnology to ensure only safe and effective strains are utilized. Functional probiotic properties are strain-specific. Therefore, the future of enterococcal probiotics in clinical use is delicate and requires balancing the exploitation of their multifaceted therapeutic and antimicrobial potential across multiple infectious and non-infectious diseases with the successful management of associated risks. Future research should focus on the high-resolution genomic and functional characterization of emerging and less-studied *Enterococcus* species, the development of precision tools such as CRISPR-Cas systems for targeted removal of undesirable traits, and the exploration of synthetic biology approaches to engineer safer, customized probiotic strains. Such efforts will be essential in harnessing the full potential of *Enterococcus* spp. while enhancing food security and safeguarding public health.

## Data Availability

No new data were created or analyzed in this study.
